# Overcoming Ferroptosis-Induced Exhaustion of NK Cells through Inhibition of the ATF3-Mediated Integrated Stress Response in Ovarian Cancer

**DOI:** 10.7150/ijbs.112615

**Published:** 2025-08-30

**Authors:** Tian Chen, Shulin Zhou, Yashuang Zhang, Huangyang Meng, Huixian Miao, Mingming Feng, Yi Jiang, Yicong Wan, Lin Zhang, Wenjun Cheng

**Affiliations:** Department of Gynecology, the First Affiliated Hospital of Nanjing Medical University, Nanjing 210029, Jiangsu, China.

**Keywords:** NK cell, ovarian cancer, ferroptosis, NRF2, integrated stress response, tumor microenvironment

## Abstract

The absence of cytotoxic effector cells, such as CD8⁺ T cells or Natural Killer (NK) cells, within tumors establishes an immune-cold tumor microenvironment (TME), contributing to poor immunotherapy responses, as observed in ovarian cancer. Although prior studies implicate NK cell exhaustion within the TME related to ferroptosis, the underlying mechanisms remain undefined. This study demonstrates that upon infiltrating the ovarian cancer TME, NK cells activate an integrated stress response (ISR) centered on ATF3. This ATF3-mediated ISR suppresses NRF2 expression, compromising their ability to counteract oxidative stress and ultimately triggering ferroptosis. Critically, we show that co-treatment with the ISR inhibitor ISRIB and NK cells not only prevents NK cell ferroptosis but also synergizes to enhance tumor cell killing. These findings provide novel insights into the mechanisms driving NK cell exhaustion within the TME and identify ISR inhibition as a promising therapeutic target and intervention strategy for developing NK cell-based therapies against ovarian cancer.

## Introduction

Ovarian cancer (OC) is the most lethal gynecologic malignancy worldwide due to its high rates of drug resistance and recurrence; its 5-year survival rate is only approximately 40%; thus, the development of novel therapies is urgently needed. However, OC is a typical immunosuppressive tumor, and thus far, the results of clinical trials of OC immunotherapy have not been satisfactory 1, 2. Previous immunotherapies for OC were based on immune checkpoint inhibitors. As the field of immunotherapy has advanced, adoptive cellular immunotherapy has emerged as a promising new treatment for OC. Compared with T cells, natural killer (NK) cells are ideal target cells for next-generation immunotherapies, as they have a broader response range, stronger antitumor effects, and fewer side effects 3-5. However, the poor infiltration of NK cells and their short duration of viability and activity in tumors hinder their application in patients with solid (nonhematological) tumors 6, 7.

Research has shown that increased lactate levels in metastatic colorectal cancer cells in the liver can induce apoptosis in liver-resident NK cells 8. In addition, L-kynurenine, the central metabolite of tryptophan degradation catalyzed by indoleamine 2,3-dioxygenase, has been found to have proapoptotic effects on NK cells 9. Moreover, another study reported that the main form of death associated with L-kynurenine-induced NK cell loss in gastric cancer is ferroptosis 10. Although these studies focused on the phenomenon of decreased NK cell activity in tumors, the mechanism of NK cell depletion in the TME of OC is still unclear.

In environments with complex stresses, such as genomic instability, oxidative stress and nutrient deficiencies, sensing kinases induce phosphorylation of the α-subunit of EIF2, leading to a decrease in the rate of general translation initiation but an increase in the translation of specific mRNAs, such as those of ATF4, ATF3, and CHOP, to facilitate cell survival; these events are collectively called the integrated stress response (ISR). An ISR that is too weak or too strong can lead to cell death. Therefore, it is widely believed that, owing to various complex stresses within the TME, the ISR within tumor cells remains at its optimal state. On this basis, the ISR inhibitor ISRIB was found to kill tumor cells, such as pancreatic ductal adenocarcinoma cells 11, 12. When NK cells were cocultured with OC cells, we observed unique alterations in the morphology and functionality of NK cells, which piqued our interest in their fate in the tumor microenvironment (TME) and prompted us to assess the dynamic changes in their metabolic patterns and stress response that led to a poor outcome.

In this study, we found that when NK cells enter the TME, which is characterized by the coexistence of complex stimuli such as abundant reactive oxygen species (ROS), NK cells activate the ISR centered on ATF3, thereby inhibiting NRF2 and preventing them from combating NCOA4-mediated iron overload, ultimately leading to ferroptosis induction. Furthermore, we found that the combination of the ISR inhibitor ISRIB with NK cell therapy not only enabled direct targeting OC cells but also indirectly increased the cytotoxic effect of NK cells on OC cells by suppressing their ferroptosis, providing a new strategy for the application of NK cells in solid tumor treatment.

## Materials and methods

### Analysis of public expression data

In this study, we utilized the GSE184880 dataset from the public Gene Expression Omnibus database and performed single-cell RNA sequencing data analysis via the R Studio server with Seurat version 5. Initially, cells were subjected to quality filtering on the basis of the percentage of mitochondrial, exogenous, and ribosomal genes expressed; therefore, low-quality cells, certain confounding factors, and cellular chimeras were eliminated. The data were subsequently subjected to normalization and standardization processes, followed by principal component analysis. Dimensionality reduction was carried out using t-distributed stochastic neighbor embedding and uniform manifold approximation and projection for preliminary clustering of cells. Potential batch effects were examined among samples, and batch integration was performed using the Harmony algorithm. Clusters were formed at various resolutions on the basis of the visualization of cell clustering, and an appropriate resolution was selected for clustering. The FindAllMarkers algorithm was used to identify differentially expressed genes across all clusters, and cells were annotated on the basis of these differentially expressed gene and cell-specific marker genes. Data visualization techniques were applied to present the findings. P adj < 0.01 was considered to indicate statistical significance. The R packages/toolkits used in the study included dplyr, tidyverse, Seurat, Matrix, GEOquery, future, clustree and ggrepel.

### Patients and specimens

This study was approved by the Ethics Committee of the First Affiliated Hospital of Nanjing Medical University (2020-MD-061.A1). Normal ovarian tissue samples (n = 10) and epithelial OC tissue samples (n = 10) were collected from newly diagnosed OC patients with informed consent. The surgical pathological and clinical data of the patients were previously published in another article by our team 13. OC patient-derived organoids (PDOs) were established according to the foundational work of our research group 14. The peripheral blood used for NK cell extraction was voluntarily provided by healthy researchers.

### IF staining

Paraffin-embedded tissue sections were subjected to TSA-mIF staining according to the protocol of the Quadruple Fluorescence Immunohistochemistry Mouse/Rabbit Kit (ImmunoWay Biotechnology Company, USA). For IF staining, NK cells were incubated overnight with primary antibodies after fixation, permeabilization, and blocking and were then incubated with fluorescently labeled secondary antibodies for 1 h. Images were acquired using a 20X objective on a laser scanning microscope (LSM900 with Airyscan2, Zeiss Microscopy, Germany). The primary antibodies used in this study are listed in Table S1.

### Cell culture

The HEK293T cell line, human OC cell lines (SKOV3, OVCAR3, HO-8910, and A2780), and human NK cell line (YT) used in this study were obtained from the American Type Culture Collection, authenticated by shot tandem repeat profiling, and regularly tested for mycoplasma contamination. The cells were cultured at 37 °C with 5% CO_2_. HEK293T cells were cultured in Dulbecco's modified Eagle's medium (DMEM; Gibco, USA) supplemented with 10% fetal bovine serum (FBS; Gibco, USA) and 1% penicillin‒streptomycin (Procell, China). The human OC cell lines SKOV3, OVCAR3, HO8910, and A2780 and the human NK cell line YT were cultured in Roswell Park Memorial Institute 1640 medium (supplemented with 10% FBS and 1% penicillin‒streptomycin). In accordance with the manufacturer's instructions, NK cells were isolated from peripheral blood using a PBMC isolation kit (Fcmacs, China) and a human NK cell efficient amplification kit (StemEry, China) and cultured *in vitro*. Three coculture systems, namely, direct coculture, indirect coculture, and conditioned culture, were used in this study. In the indirect coculture system, OC cells were seeded in the lower chambers of a 6-well plate (Corning, USA) with 0.4 μm Transwell® membrane inserts (Corning, USA), and NK cells were added to the upper chambers. In the conditioned culture system, medium from OC cells cultured for 36 hours was collected, centrifuged and filtered through a sterile 0.22 μm filter to eliminate residual cells. The filtered medium supplemented with 10% FBS served as the culture medium for the conditioned culture of the NK cells. In the coculture system of NK cells and OC PDOs, NK cells were mixed with OC PDOs, and Matrigel was added at a final concentration of 70%. The mixture was subsequently seeded into 96-well plates.

### Flow cytometry

Flow cytometry was utilized to assess the expression of cell surface antigens, cell viability (7-aminoactinomycin D dye, Thermo Fisher Scientific, USA), cell proliferation ability (eFluor™ 670 proliferation dye, Thermo Fisher Scientific, USA), intracellular ROS levels (2',7'-dichlorodihydrofluorescein diacetate dye, Beyotime, China), and lipid peroxidation levels (lipid peroxidation sensor BODIPY 581/591 C11, Thermo Fisher Scientific, USA) according to the manufacturers' instructions. The lipid peroxidation level was quantified as the ratio of the mean fluorescence intensity at 590 nm to that at 510 nm; a lower ratio indicates a higher lipid peroxidation level. Flow cytometry was performed on an LSRFortessa cytometer (BD Biosciences). The data were analyzed using FlowJo_V10.

### Cellular cytotoxicity and proliferation assay

Cell viability was evaluated using a Lactate Dehydrogenase Release Assay Kit (Beyotime, China) and a Cell Counting Kit-8 (Beyotime, China) according to the instructions. The Cell Counting Kit-8 assay was utilized to evaluate the cytotoxic effect of NK cells on OC cells after conditioned culture.

### Transcriptome analysis

Total RNA was extracted from samples in both the control and conditioned culture groups using an RNeasy kit (Beyotime, China), and quality control was performed on the extracted RNA using a Qubit 4.0 fluorometer. After removing ribosomal RNA using oligo(dT) probes, libraries were constructed using the Ultra™ II Directional RNA Library Prep Kit for Illumina (E7765S, NEB, MA) and then sequenced on the Illumina HiSeq X Ten sequencing platform at Novogene Biotechnology, LLC. The raw sequence data reported in this paper have been deposited in the Genome Sequence Archive (Genomics, Proteomics & Bioinformatics 2021) of the National Genomics Data Center (Nucleic Acids Res 2022), China National Center for Bioinformation/Beijing Institute of Genomics, Chinese Academy of Sciences (GSA-Human: HRA007605) and are publicly accessible at https://ngdc.cncb.ac.cn/gsa-human.

### MDA and GSH assays

The MDA level was measured according to the instructions provided with the Lipid Peroxidation MDA Assay Kit (Beyotime, China), and the amount of MDA per unit of protein was calculated. The GSH level was measured according to the instructions provided with the GSH and GSSG Assay Kit (Beyotime, China).

### Protein extraction and Western blotting

Proteins were extracted using radioimmunoprecipitation assay buffer (Sigma‒Aldrich, USA) containing protease and phosphatase inhibitors (Beyotime, China), and the protein concentration was quantified using an Enhanced BCA Protein Assay Kit (Beyotime, China). Proteins were separated by 10% sodium dodecyl sulfate‒polyacrylamide gel electrophoresis and then transferred onto polyvinylidene fluoride membranes. The membranes with bound proteins were incubated sequentially with primary and secondary antibodies, and protein expression was visually evaluated via enhanced chemiluminescence (Thermo Fisher, USA).

### Cell transduction

Through a sequence of procedures, including plasmid extraction, lentiviral packaging, lentiviral infection, and selection, the firefly luciferase gene sequence was transduced into SKOV3 cells. Through this protocol, a SKOV3 cell line with stable expression of the luciferase gene was established. This luciferase-expressing SKOV3 cell line was utilized as an investigative tool in animal studies to monitor tumor growth and alterations in murine models.

### Animal experiments

The animal study protocols employed in this research were designed in accordance with the "Guidelines for the Care and Use of Experimental Animals" and were approved by the Animal Ethics Committee of Nanjing Medical University (IACUC-2207012). Six- to eight-week-old athymic nude mice weighing 16 to 18 g were selected. Abdominal OC tumors were established in mice via the intraperitoneal injection of luciferase-labeled SKOV3 cells (10^6^ cells/100 μL). After successful establishment of the model, the mice were randomly divided into groups for different treatments. *In vivo* imaging was conducted to evaluate the abdominal tumor burden in the mice in the different treatment groups. After isolation from the peritoneal lavage fluid, the lymphocytes were separated using a lymphocyte separation kit, and the relevant indicators of NK cells labeled with specific dyes were quantified by flow cytometry. The infiltration of NK cells into the OC tumor core after different treatments was evaluated via TSA-mIF staining.

### RNA isolation and qRT‒PCR analysis

RNA was extracted using the TRIzol method prior to quantification. The extracted RNA was then reverse transcribed into cDNA using a reverse transcription kit (Vazyme, China). qRT‒PCR was conducted on a LightCycler® 480 Instrument II (Roche, Switzerland) employing SYBR Green qPCR Master Mix (Vazyme, China). GAPDH served as an internal reference. The sequences of the primers used for qRT‒PCR are listed in Table S2.

### Statistical analysis

Statistical analyses of the experimental data were conducted using GraphPad Prism 10 software, with a minimum of three replicates per experiment. For comparisons between two groups, the two-tailed unpaired t test was used, whereas one-way ANOVA with Dunnett's post hoc test was used for comparisons between a control group and multiple treatment groups. Differences with a P value of less than 0.05 were considered statistically significant, and the degree of significance was denoted *: P < 0.05, **: P < 0.01, and ***: P < 0.001.

## Results

### Low NK infiltration in the TME of OC is due to decreased NK cell viability

**Figure 1:**
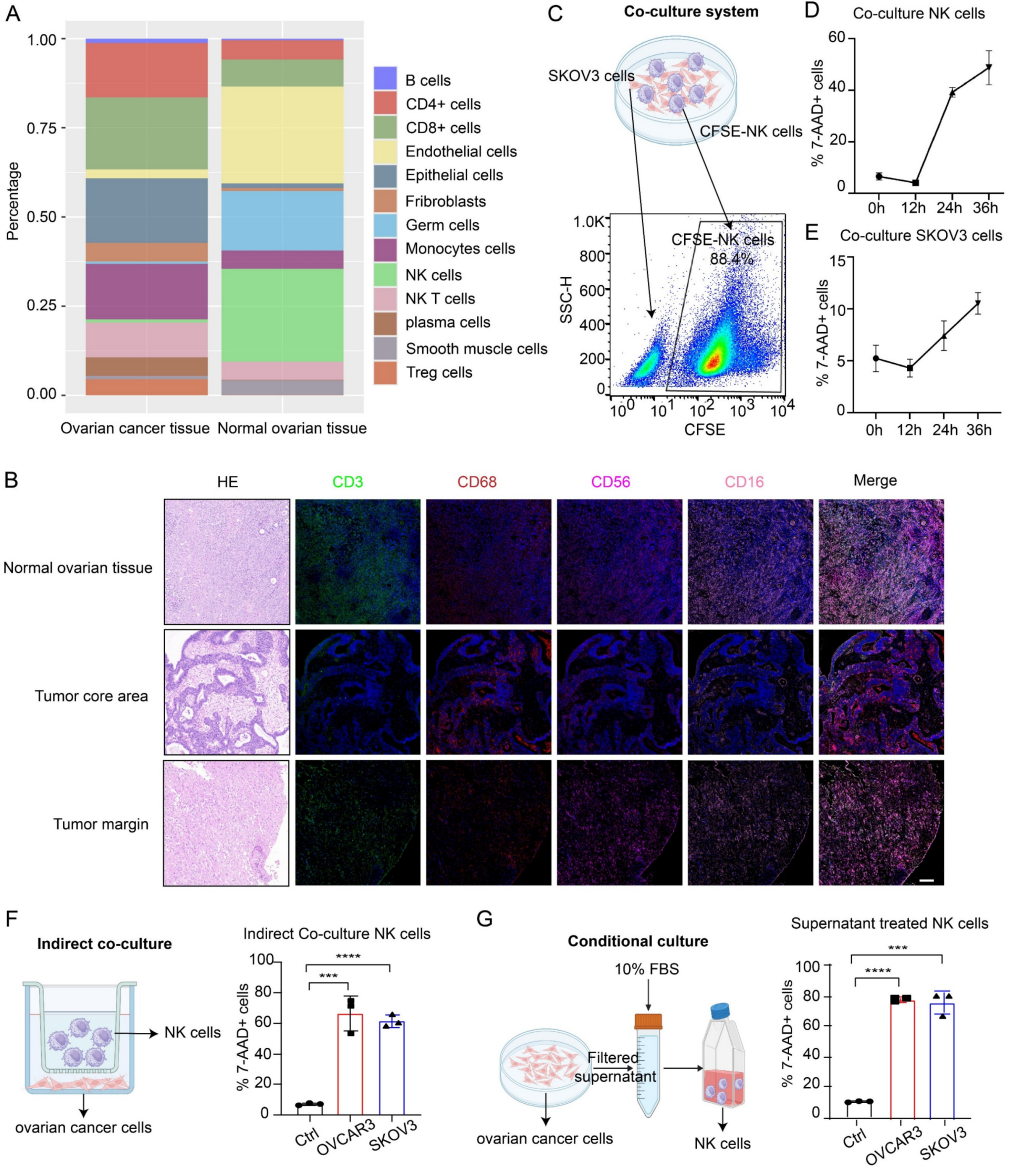
** Low NK cell infiltration in the TME of OC is due to decreased NK cell viability.** (A) Cell composition ratio graph of the OC tissue group (n=7) and the normal ovarian tissue group (n=5) from the GSE184880 dataset. (B) Representative TSA-mIF images of normal ovarian tissues (n=10), the core area of OC tissues (n=10) and the margin area of OC tissues (n=10). Nuclei, 4,6-diamidino-2-phenylindole (DAPI); CD3, green; CD68, red; CD56, rose; CD16, pink. CD3+: T cells, CD56+CD16+: NK cells, CD68+: macrophages. Scale bar, 100 µm. (C) Schematic diagram of the direct coculture system of ovarian cancer cells and CFSE-labeled NK cells and the method for separate detection of indicators of ovarian cancer cells and NK cells by flow cytometry. (D) Proportion of CFSE-labeled NK cells in the 7AAD+ group at 12-hour increments after direct coculture; n=3. (E) Proportion of 7AAD+ SKOV3 cells in each group at 12-hour increments after direct coculture; n=3. (F) Proportion of 7AAD+ NK cells after indirect coculture with OVCAR3 and SKOV3 cells for 36 hours, n=3. (G) Proportion of 7AAD+ NK cells after 36 hours of conditional culture with the supernatants from OVCAR3 and SKOV3 cell culture media; n=3. The error bars represent the SEM.

Through analysis of the GSE184880 dataset from the Gene Expression Omnibus (GEO) database, we found a notable reduction in the proportion of NK cells within OC tissues compared with that in normal ovarian tissues (Figure S1A-B, Figure 1A). TSA-mIF staining of normal ovarian tissues and epithelial OC tissues collected from our center revealed significantly diminished levels of CD3-labeled T cells and CD56- and CD16-labeled NK cells in OC tissues, particularly in the tumor core, alongside abundant infiltration of CD68-labeled macrophages (Figure 1B). These findings underscore the decreased abundance of NK cells in OC. Given the dense vascularity of tumors and the propensity for macrophage infiltration, we posited that the low infiltration of NK cells in OC was due to decreased viability of these cells. To investigate the effects of OC cells on NK cells and the underlying mechanism, we employed three culture models for the experiments. In direct coculture experiments, among NK cells prelabeled with CFSE dye, 50% died within 36 hours; only 10% of OC cells died within this period (Figure 1C-E), indicating compromised NK cell viability and tumoricidal activity in the TME of OC. In the indirect coculture model, chambers were used to separate the two types of cells to prevent them from coming into direct contact, and the proportion of dead NK cells in the experimental group was still significantly greater than that in the control group after 36 hours (Figure 1F). In conditional culture assays using OC cell culture medium, the proportion of dead NK cells was also higher than that in the control group (Figure 1G), as corroborated by lactate dehydrogenase (LDH) release assays (Figure S1C) and Cell Counting Kit-8 (CCK-8) assays (Figure S1D), which collectively demonstrated diminished NK cell viability following exposure to the OC TME without cell‒cell interactions. In PDOs, the proportion of dead NK cells also increased (Figure S1E), and these cells exhibited decreased tumor killing ability after coculture (Figure S1F). These results indicate that NK cells undergo extensive cell death upon entering the TME of OC, thereby affecting their ability to exert antitumor effects.

To eliminate potential confounding factors, we evaluated the proliferation, differentiation and activation of NK cells. First, we observed a reduction in the mitotic index of NK cells following conditional culture (Figure S2A). In addition, there was no significant difference in differentiation between the conditional culture group and the control group (Figure S2B), and 20% of the NK cells were activated (Figure S2C). The impairments in the cytotoxic and proliferative abilities of NK cells in the TME of OC may be due to their decreased viability. However, owing to the predominant presence of the CD16-positive cluster of NK cells utilized in our study and the absence of appropriate real-world controls, these findings may not accurately reflect the actual dynamics of NK cell differentiation and activation within the OC TME.

### NK cells entering the TME of OC undergo ferroptosis

**Figure 2:**
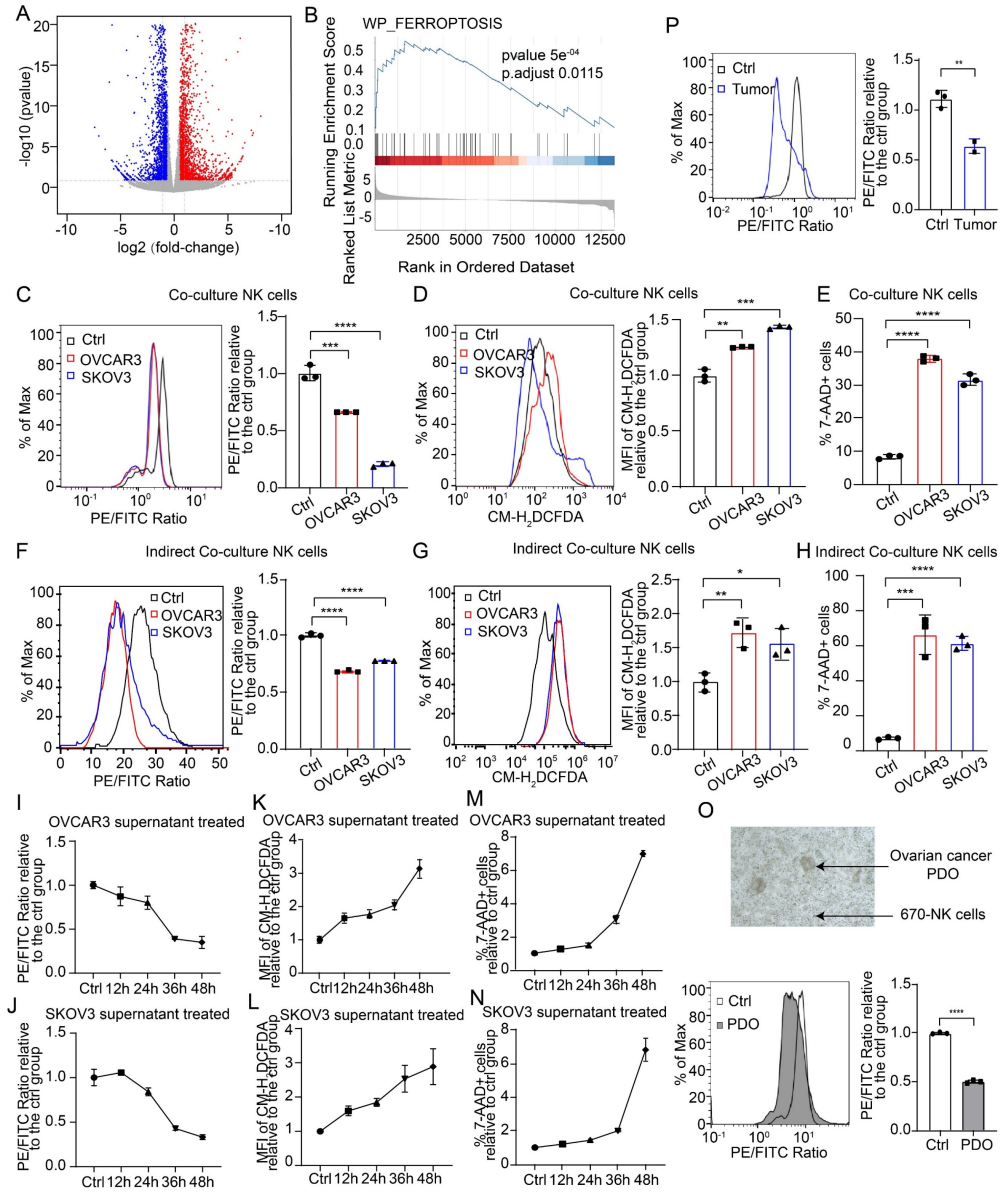
** NK cells entering the TME of OC undergo ferroptosis.** (A) Volcano plot of differentially expressed genes in NK cells from the control and SKOV3 conditional culture groups, n=3. Significant genes are highlighted in either red (upregulated in the conditional culture group) or blue (downregulated in the conditional culture group). (B) GSEA enrichment score of differentially expressed genes in the ferroptosis pathway. (C) Lipid peroxidation level of 670 labeled NK cells after direct coculture with OVCAR3 and SKOV3 cells for 36 hours, n=3. (D) ROS levels in 670 labeled NK cells after direct coculture with OVCAR3 and SKOV3 cells for 36 hours, n=3. (E) Proportion of 7AAD-positive CFSE-labeled NK cells after direct coculture with OVCAR3 and SKOV3 cells for 36 hours; n=3. (F) Lipid peroxidation levels of 670 labeled NK cells after indirect coculture with OVCAR3 and SKOV3 cells for 36 hours, n=3. (G) ROS levels in 670 labeled NK cells after indirect coculture with OVCAR3 and SKOV3 cells for 36 hours, n=3. (H) Proportion of 7AAD-positive CFSE-labeled NK cells after indirect coculture with OVCAR3 and SKOV3 cells for 36 hours; n=3. (I) Lipid peroxidation level of NK cells at 12-hour increments after conditional culture with the supernatant from OVCAR3 cell culture media, n=3. (J) Lipid peroxidation level of NK cells at 12-hour increments after conditional culture with the supernatant from SKOV3 cell culture media; n=3. (K) ROS levels in NK cells at 12-hour increments after conditional culture with the supernatant from OVCAR3 cell culture media, n=3. (L) ROS levels of the NK cells at 12-hour increments after they were subjected to conditional culture with the supernatant from the SKOV3 cell culture media; n=3. (M) Proportion of 7AAD-positive NK cells at 12-hour increments after conditional culture with the supernatant from OVCAR3 cell culture media; n=3. (N) Proportion of 7AAD-positive NK cells at 12-hour increments after conditional culture with the supernatant from the SKOV3 cell culture media; n=3. (O) Lipid peroxidation levels of 670 labeled NK cells after direct coculture with OC PDOs for 36 hours, n=3. (P) Lipid peroxidation levels of 670 labeled NK cells in the peritoneal lavage fluid of control (n=3) and OC abdominal tumor-bearing (n=2) mice. The error bars represent the SEM.

To investigate the mechanism of impaired NK cell viability in the TME of OC, we conducted transcriptome sequencing on NK cells from both the control and conditional culture groups, followed by functional enrichment analysis of the differentially expressed genes (Figure 2A, Figure S3A-B). Gene set enrichment analysis revealed that the differentially expressed genes were significantly enriched in the ferroptosis pathway (Figure 2B), and the heatmap revealed significant differences in the expression of genes involved in the ferroptosis pathway between the control group and the conditional culture group (Figure S3C). These findings suggest that NK cells in the OC TME may undergo ferroptosis. We also used direct coculture, indirect coculture and conditional culture models to assess whether the form of NK cell death was ferroptosis. After 36 hours of direct coculture with OC cells, the lipid peroxidation level in NK cells was significantly elevated (Figure 2C), which was determined by the ratio of reduced to oxidized lipids, with lower ratios indicating higher levels of lipid peroxidation. In addition, the ROS level (Figure 2D) and mortality rate (Figure 2E) of NK cells were significantly increased after direct coculture, indicating that direct coculture with OC cells triggers ferroptosis in NK cells. A similar phenomenon was observed in the indirect coculture model, where NK cells exhibited increased lipid peroxidation (Figure 2F), ROS levels (Figure 2G), and cell death (Figure 2H) after 36 h, indicating that OC cells can induce ferroptosis in NK cells even without direct physical contact. Moreover, when NK cells were conditionally cultured with OC cell supernatant for increasing durations, the lipid peroxidation level (Figure 2I-J), ROS level (Figure 2K-L), and proportion of dead cells (Figure 2M-N) gradually increased, indicating that NK cells undergo ferroptosis due to conditions in the TME of OC. The level of malondialdehyde (MDA), a lipid peroxidation product, was elevated (Figure S3D), the intracellular reduced glutathione (GSH) level was decreased (Figure S3E), and the expression of PTGS2, a marker of ferroptosis, was increased in NK cells in the conditional culture model (Figure S3F), further verifying that NK cells in the TME of OC undergo ferroptosis. Furthermore, NK cells cocultured with OC PDOs presented increased lipid peroxidation levels (Figure 2O). In a mouse model with abdominal OC tumors, intravenous administration of eFluor™ 670-labeled NK cells resulted in significantly higher lipid peroxidation levels in NK cells isolated from ascites than in those isolated from peritoneal lavage fluid from nontumor-bearing mice (Figure 2P). These data prove that OC cells can induce the ferroptosis of NK cells by altering the TME.

### Ferroptosis inhibitors inhibit NK cell death and restore the tumoricidal effects of NK cells

**Figure 3:**
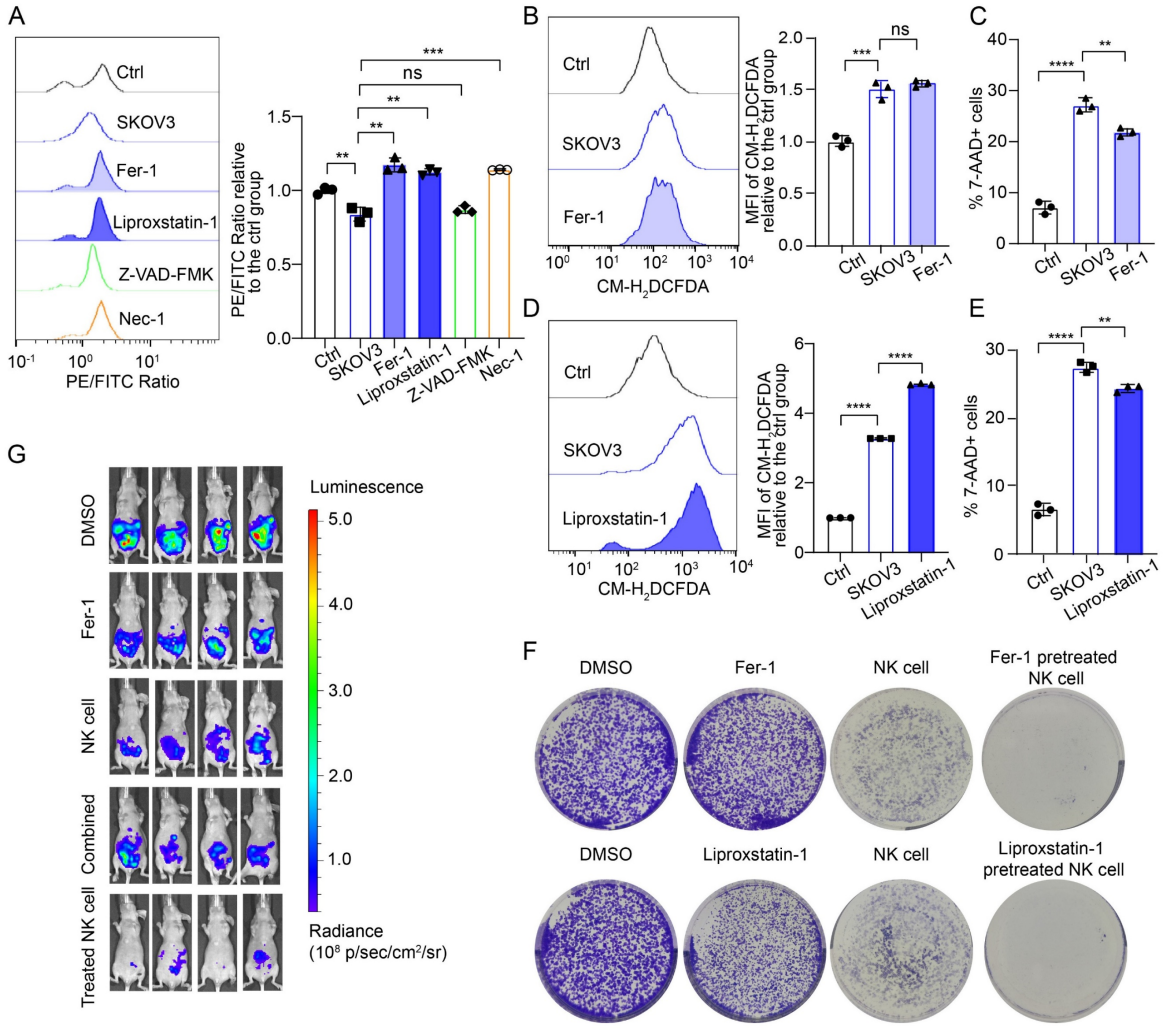
** Ferroptosis inhibitors inhibit the ferroptosis of NK cells and restore their tumoricidal effect.** (A) Lipid peroxidation level in NK cells after 36 hours of culture with the supernatant from SKOV3 cell culture media, followed by pretreatment with Fer-1 (10 μM), liproxstatin-1 (10 μM), Z-VAD-FMK (10 μM) or Nec-1 (10 μM) for 4 hours, n=3. (B) ROS levels in NK cells after 36 hours of culture with the supernatant from SKOV3 cell culture media, followed by pretreatment with Fer-1 (10 μM) for 4 hours, n=3. (C) Proportion of 7-AAD-positive NK cells after 36 hours of culture with the supernatant from the SKOV3 cell culture media, followed by pretreatment with Fer-1 (10 μM) for 4 hours, n=3. (D) ROS levels in NK cells after 36 hours of culture with the supernatant from SKOV3 cell culture media, followed by pretreatment with liproxstatin-1 (10 μM) for 4 hours, n=3. (E) Proportion of 7-AAD-positive NK cells after 36 hours of culture with the supernatant from the SKOV3 cell culture media, followed by pretreatment with liproxstatin-1 (10 μM) for 4 hours, n=3. (F) Effects of NK cells, ferroptosis inhibitors and NK cells pretreated with ferroptosis inhibitors on the colony formation ability of SKOV3 cells treated with 10 μM Fer-1 or 10 μM liproxstatin-1. (G) Fluorescence images showing the tumor burden in ovarian cancer abdominal tumor-bearing mice treated with Fer-1, NK cell therapy, combined Fer-1 and NK cell therapy, or NK cell therapy with Fer-1 pretreatment. The error bars represent the SEM.

To further confirm that ferroptosis is the mechanism of NK cell death in the OC TME, we used two commonly used ferroptosis inhibitors, ferrostatin-1 (Fer-1) and liproxstatin-1, along with an apoptosis inhibitor (ZVAD-FMK) and a necrosis inhibitor (necrostatin-1), to pretreat NK cells. The lipid peroxidation level of the NK cells pretreated with either the ferroptosis inhibitor or the necrosis inhibitor was lower than that of the untreated cells, whereas pretreatment with the apoptosis inhibitor did not decrease the lipid peroxidation level of the NK cells (Figure 3A; Figure S4A, S4D). The cross-regulatory relationship between necroptosis and ferroptosis has been discussed in other studies but was not further investigated in this study 15, 16. Ferroptosis inhibitor treatment did not decrease ROS levels in NK cells (Figure 3B, 3D; Figure S4B, S4E), which may be associated with the mechanism of ferroptosis inhibitors. Importantly, ferroptosis inhibitor treatment reduced the proportion of dead NK cells in conditional culture (Figure 3C, 3E; Figure S4C, S4F).

To evaluate whether pretreatment with ferroptosis inhibitors can restore the cytotoxic effect of NK cells on OC cells, we conducted long-term direct coculture and observation of OC and NK cells. Initially, NK cells demonstrated cytotoxic effects on OC cells; however, over a prolonged coculture period, OC cells gradually exhibited dispersive migratory behavior, and the proportion of dead NK cells increased. As the number of viable NK cells decreased, the growth advantage of the surviving OC cells was restored. Colony formation assays revealed that ferroptosis inhibitors alone did not induce the death of OC cells; however, pretreatment with ferroptosis inhibitors increased the proportion of viable NK cells in the OC TME and increased their cytotoxic effects on OC cells (Figure 3F, Figure S4G). We established abdominal OC tumors in mice with SKOV3 cells, treated them with Fer-1, NK cells, Fer-1 combined with NK cells, or Fer-1-pretreated NK cells at 7 different time points, and then performed *in vivo* imaging. Treatment with Fer-1 alone did not significantly affect tumor growth, whereas treatment with NK cells alone was effective in eliminating OC cells. Interestingly, combining Fer-1 with NK cells did not enhance NK cell cytotoxicity against OC cells. In contrast, mice treated with Fer-1-pretreated NK cells presented minimal abdominal tumor burden (Figure 3G). In addition, there was no significant weight loss in the NK cell-treated group of mice, suggesting the safety of the treatment (Figure S4H). These findings suggest that ferroptosis inhibitor pretreatment can enhance the tumoricidal effect of NK cells, while the dysfunction of combination therapy may result from two factors: the previously reported protective effect of ferroptosis inhibitors on tumor cells and the rapid induction of ferroptosis in NK cells after they enter the OC TME.

### NK cells in the TME of OC undergo ferroptosis due to the inhibition of NRF2 expression

**Figure 4:**
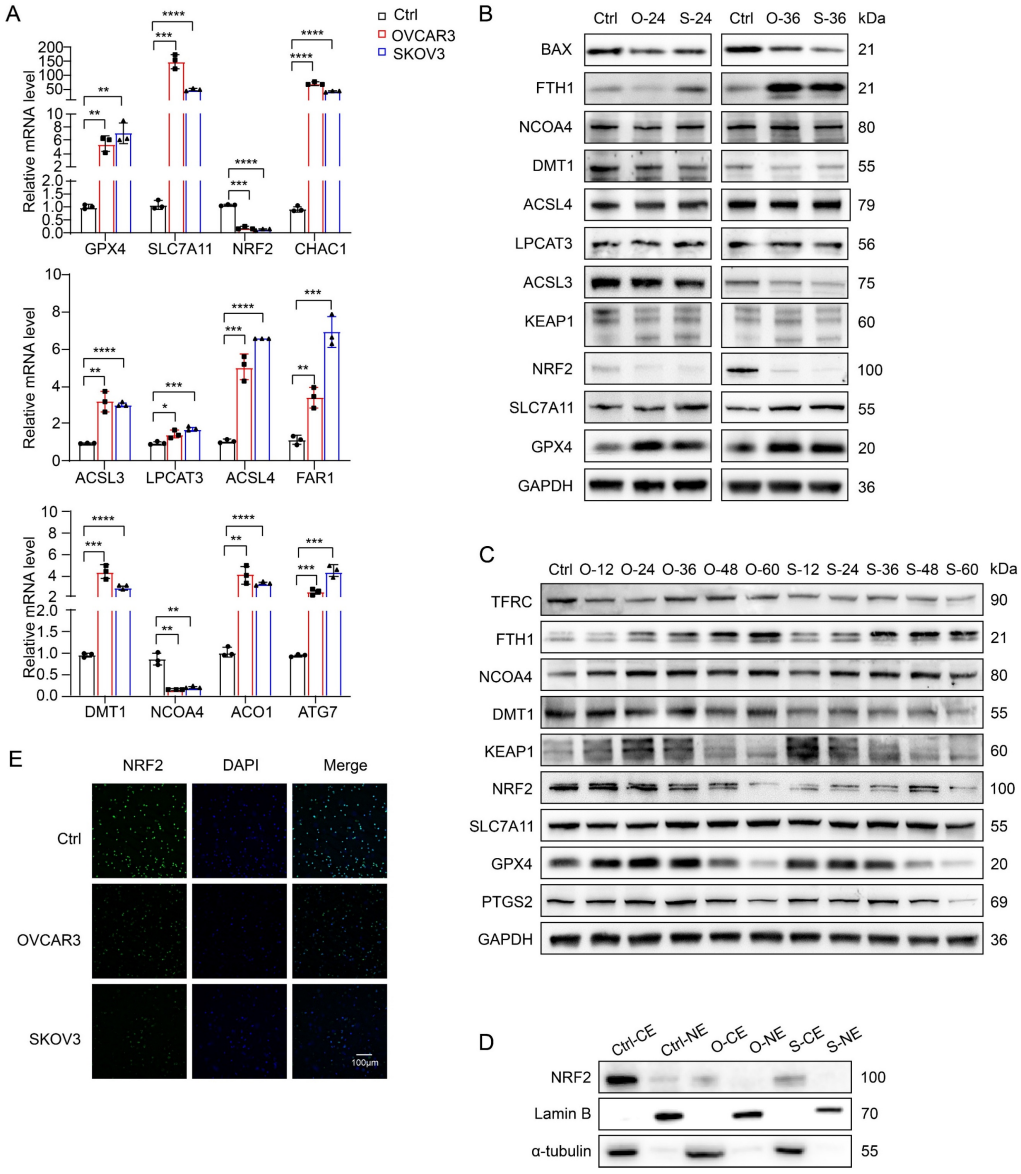
** NK cells in the TME of OC undergo ferroptosis due to the inhibition of NRF2 expression.** (A) Differences in the mRNA levels of ferroptosis-related genes in NK cells between the control group and the conditional culture group with supernatants from OVCAR3 and SKOV3 cell culture media for 36 hours, n=3. (B) Differences in the protein expression of ferroptosis-related genes in NK cells between the control group and the conditional culture group with supernatants from OVCAR3 and SKOV3 cell culture media for 24 and 36 hours. (C) Differences in the protein expression of ferroptosis-related genes in NK cells between the control group and the conditional culture group with supernatants from OVCAR3 and SKOV3 cell culture media for 0, 12, 24, 36, 48, and 60 hours. (D) NRF2 expression in cytoplasmic extract (CE) and nuclear extract (NE) in NK cells from the control group and the conditional culture group after culture with supernatants from OVCAR3 and SKOV3 cell culture media for 36 hours. (E) Immunofluorescence images showing NRF2 expression in the cytoplasm and nucleus of NK cells from the control group and the conditional culture group after culture with supernatants from OVCAR3 and SKOV3 cell culture media for 36 hours. The error bars represent the SEM.

To further investigate the mechanism of ferroptosis in NK cells in the OC TME, we first used quantitative reverse transcription‒polymerase chain reaction (qRT‒PCR) to identify and compare differences in the messenger RNA (mRNA) levels of ferroptosis-related genes (FRGs) in NK cells from the conditioned culture and control groups (Figure 4A) and detected increases in the mRNA levels of most ferroptosis-inhibiting genes (FIGs) and ferroptosis-promoting genes (FPGs), such as GPX4 (FIG), SLC7A11 (FIG) and CHAC1 (FPG) in the redox pathway 17-19; ACSL3 (FIG), LPCAT3 (FPG), ACLS4 (FPG) and FAR1 (FPG) in the lipid metabolism pathway 20-23; and DMT1 (FPG), NCOA4 (FPG), ACO1 (FPG) and ATG7 (FPG) in the iron metabolism pathway 24-27. The increased mRNA levels of FPGs indicate active ferroptosis in NK cells, whereas the increased mRNA levels of FIGs suggest an antagonistic response of ferroptosis resistance in NK cells. Notably, unlike the increased mRNA level of GPX4 (FIG), the mRNA level of NRF2 (FIG) 28, a key gene that initiates the transcription of antioxidant response elements, was significantly decreased in the conditioned culture group.

We extracted proteins from NK cells cultured with OVCAR3 and SKOV3 cell supernatants for 24 and 36 hours for Western blotting and found that the expression of GPX4, SLC7A11, NCOA4, and FTH1 increased, whereas that of ACSL3, LPCAT3, and ACSL4 did not change significantly, and the expression of DMT1 and BAX decreased (Figure 4B). These results suggest that lipid metabolism pathways may not be the key pathways leading to the ferroptosis of NK cells, whereas redox and iron metabolism pathways may be involved in regulating ferroptosis in NK cells. Notably, the NRF2 protein level was significantly decreased, which was consistent with the change in the NRF2 mRNA level. To determine whether NRF2 is the key factor driving ferroptosis in NK cells within the OC TME, we performed long-term conditioned culture and analyzed protein expression in NK cells at 12-hour intervals (Figure 4C). With increasing conditioned culture time, the expression of PTGS2 gradually increased, peaking at 36 hours. Prior to the onset of ferroptosis, there was an increase in GPX4 and SLC7A11 expression, indicative of an initial adaptive response by NK cells to counter ferroptosis. Notably, NRF2 expression initially decreased at 24 hours in the OVCAR3 group and at 12 hours in the SKOV3 group, suggesting that NRF2 plays a pivotal role in driving ferroptosis in NK cells. The increased expression of KEAP1 explained the inhibition of NRF2 expression. The increased expression of NCOA4, a key protein that mediates ferritinophagy, suggests an increase in iron content in the labile iron pool, one of the upstream mechanisms of ferroptosis induction 29. It has been reported that cancer-associated fibroblasts upregulate the expression of NCOA4 in NK cells through the DIP2A-P38 pathway via a mechanism driven by cancer-associated fibroblast-derived FSTL1, leading to ferritinophagy and ferroptosis in NK cells 30. Decreased TFRC expression indicates a reduced cellular iron uptake capacity, and increased FTH1 expression can help NK cells isolate redox-active iron, which might constitute the antagonistic cellular response induced by NCOA4-mediated iron overload. Furthermore, both western blot (Figure 4D) and immunofluorescence (Figure 4E) images showed a decrease in NRF2 expression in the cytoplasm and nucleus of the treatment group, indicating a reduction in NRF2 nuclear translocation after conditional culture. On the basis of the above results, the depletion of NK cells in the OC TME is due to the rapid inhibition of NRF2 expression after NK cells enter the TME of OC, which prevents them from resisting NCOA4-mediated iron overload and ultimately leads to ferroptosis.

### TBHQ inhibits the ferroptosis of NK cells in the TME of OC and increases their cytotoxicity against OC cells

**Figure 5:**
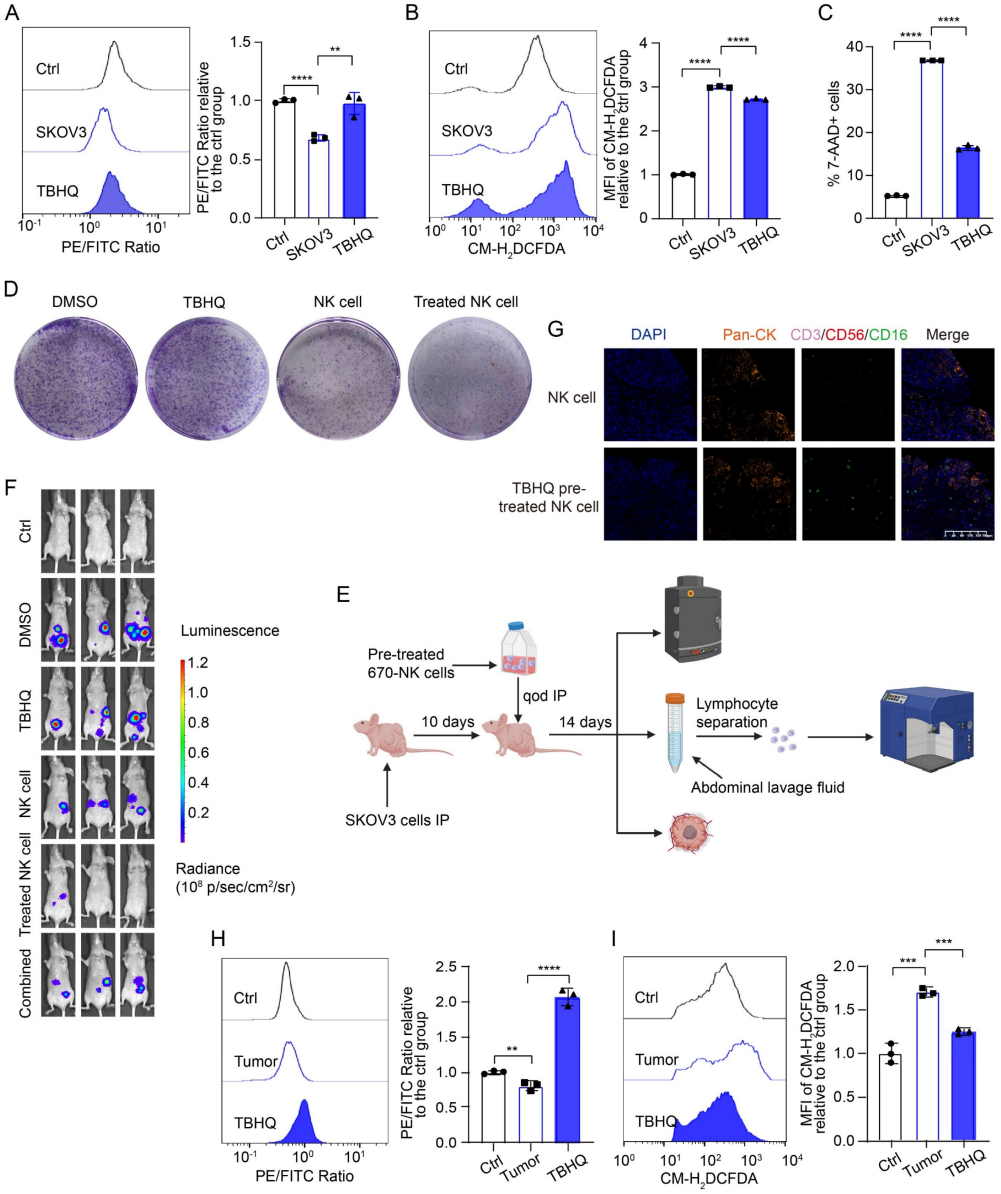
** TBHQ inhibits the ferroptosis of NK cells in the TME of OC and enhances their cytotoxic effects against OC cells.** (A) Lipid peroxidation level in NK cells after 36 hours of conditional culture with the supernatant from SKOV3 cell culture media, followed by pretreatment with TBHQ (5 μM) for 4 hours, n=3. (B) ROS levels in NK cells after 36 hours of culture with the supernatant from SKOV3 cell culture media, followed by pretreatment with TBHQ (5 μM) for 4 hours, n=3. (C) Proportion of 7-AAD-positive NK cells after 36 hours of culture with the supernatant from the SKOV3 cell culture media, followed by pretreatment with TBHQ (5 μM) for 4 hours, n=3. (D) Effects of TBHQ, NK cells and TBHQ-pretreated NK cells on the colony formation ability of SKOV3 cells. (E) Animal model diagram illustrating the enhancement of NK cell cytotoxicity with TBHQ pretreatment, which was created with BioRender. (F) Fluorescence images showing the tumor burden in OC abdominal tumor-bearing mice treated with TBHQ, NK cells, TBHQ-pretreated NK cells and combined TBHQ and NK cell therapy. (G) TSA-mIF staining images showing NK cell infiltration in the abdominal tumor core of mice treated with NK cells alone and those treated with TBHQ-pretreated NK cells. (H) Lipid peroxidation levels of 670 labeled NK cells from the peritoneal lavage fluid of mice in the control group without tumor formation, the NK cell group, and the TBHQ-pretreated NK cell group, n=3. (I) ROS levels in 670 labeled NK cells from the peritoneal lavage fluid of mice in the control group without tumor formation, the NK cell group, and the TBHQ-pretreated NK cell group, n=3. The error bars represent the SEM.

To identify potential targets for promoting ferroptosis resistance in NK cells and enhancing their cytotoxic effects on OC cells, we screened various ferroptosis-targeting drugs, namely, tert-butylhydroquinone (TBHQ; an NRF2 activator), pyridoxal isonicotinoyl hydrazine (PIH; a lipophilic trivalent iron chelator), acetylcysteine (a ROS inhibitor), artemisinin, α-vitamin E (a potent antioxidant), and NADPH, for rescue experiments (Figure S5A). We detected the most significant decrease in the lipid peroxidation level in NK cells in the group pretreated with TBHQ and PIH, indicating that these drugs resulted in the greatest inhibition of ferroptosis, which is consistent with the above findings indicating that NRF2 is inhibited and that NCOA4 is activated in NK cells in the OC TME. The lipid peroxidation level (Figure 5A, Figure S5B), ROS level (Figure 5B, Figure S5C) and mortality (Figure 5C, Figure S5D) of NK cells were significantly decreased after TBHQ pretreatment, which proved that TBHQ can inhibit the ferroptosis of NK cells in the OC TME.

A colony formation assay revealed that TBHQ-pretreated NK cells exhibited an enhanced ability to kill OC cells (Figure 5D, Figure S5E). We further validated the effect of TBHQ on the tumoricidal activity of NK cells at the animal level by *in vivo* imaging, TSA-mIF staining of peritoneal OC lesions, and measurement of the lipid peroxidation level of NK cells in peritoneal lavage fluid (Figure 5E). *In vivo* imaging revealed that TBHQ monotherapy was not effective, that treatment with NK cells alone reduced the abdominal tumor burden in mice, and that mice treated with TBHQ-pretreated NK cells had the lowest abdominal tumor burden, suggesting that TBHQ pretreatment increases the tumoricidal effect of NK cells. However, the combination-treated group results were similar to the ferroptosis inhibitor treatment group results (Figure 5F). TSA mIF staining of tumor lesions revealed significantly greater NK cell infiltration in abdominal OC lesions in the TBHQ-pretreated NK cell group than in the group treated with NK cells alone (Figure 5G), indicating that TBHQ pretreatment can promote the infiltration of NK cells into the OC tumor core. Subsequent flow cytometry analysis of NK cells isolated from peritoneal lavage fluid revealed that, compared with NK cells treated alone, TBHQ-pretreated NK cells presented reduced levels of lipid peroxidation (Figure 5H) and ROS (Figure 5I). Therefore, TBHQ pretreatment can promote the infiltration of NK cells into the OC tumor core by suppressing the ferroptosis of these cells, thereby increasing their cytotoxicity against OC cells. The weights of the mice in the NK cell treatment group did not significantly decrease, indicating that this treatment is safe (Figure S5F).

### High ROS levels in the TME of OC induce the ATF3-centered ISR and lead to the ferroptosis of NK cells

Since a high level of ROS is a typical feature of the TME and a key factor in inducing ferroptosis, we speculated that ROS in the TME of OC might be drivers of ferroptosis in NK cells. To assess the effect of ROS in the TME on ferroptosis in NK cells, we simulated the level of ROS in the TME with H_2_O_2_ and found that the lipid peroxidation level (Figure S6A), ROS level (Figure S6B), and the proportion of dead cells (Figure S6C) in H_2_O_2_-treated NK cells were significantly increased, indicating that ROS in the TME can lead to the ferroptosis of NK cells.

**Figure 6:**
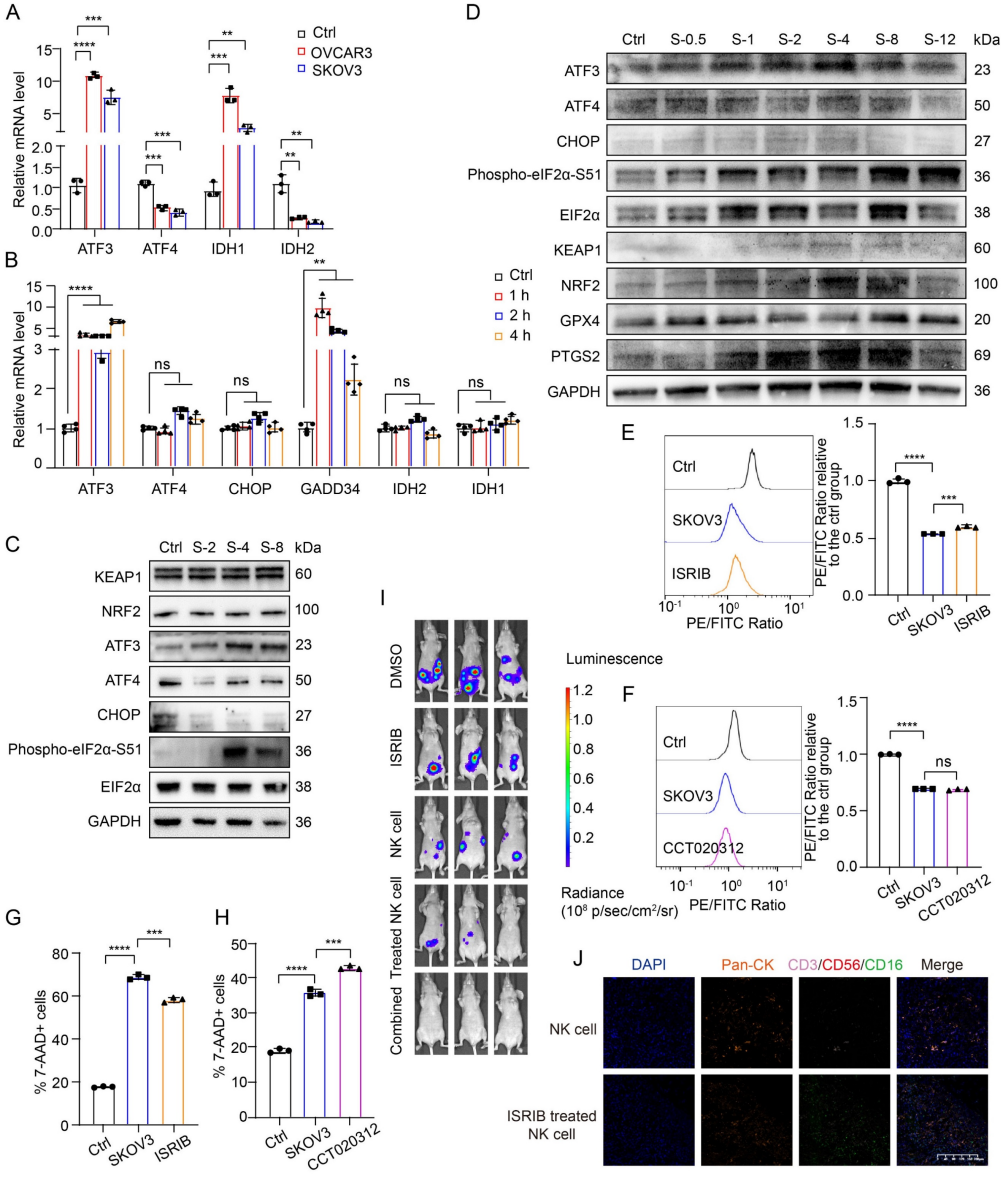
** High levels of ROS in the TME of OC activate the ATF3-mediated ISR and lead to the ferroptosis of NK cells.** (A) Differences in the mRNA levels of ISR-related genes in NK cells between the control group and the group cultured with conditioned supernatants from OVCAR3 and SKOV3 cell culture media for 36 hours, n=3. (B) Differences in the mRNA levels of ISR-related genes in NK cells between the control group and the group cultured with the conditioned medium from SKOV3 cell culture media for 1, 2 and 4 hours, n=3. (C) Differences in the protein expression of ISR-related genes in NK cells between the control group and the group cultured with the conditioned medium from SKOV3 cell culture media for 2, 4 and 8 hours. (D) Differences in the protein expression of ISR-related genes in NK cells between the control group and the group cultured with the supernatant from SKOV3 cell culture media for 0.5, 1, 2, 4, 8 and 12 hours. (E) Lipid peroxidation level in NK cells after 36 hours of conditional culture with the supernatant from SKOV3 cell culture media, followed by pretreatment with ISRIB (200 nM) for 4 hours, n=3. (F) Lipid peroxidation level in NK cells after 36 hours of conditional culture with the supernatant from SKOV3 cell culture media, followed by pretreatment with CCT020312 (10 μM) for 4 hours, n=3. (G) Proportion of 7-AAD-positive NK cells after 36 hours of culture with the supernatant from the SKOV3 cell culture media, followed by pretreatment with ISRIB (200 nM) for 4 hours, n=3. (H) Proportion of 7-AAD-positive NK cells after 36 hours of culture with the supernatant from SKOV3 cell culture media, followed by pretreatment with CCT020312 (10 μM) for 4 hours, n=3. (I) Fluorescence images showing the tumor burden in OC abdominal tumor-bearing mice treated with ISRIB, NK cells, ISRIB-pretreated NK cells and combined ISRIB and NK cell therapy. (J) TSA-mIF staining images showing NK cell infiltration into the OC abdominal tumor core of mice in the NK cell group and the ISRIB-pretreated NK cell group. The error bars represent the SEM.

To delve deeper into ISR activation in NK cells within the complex TME and its relationship with ferroptosis, we analyzed the mRNA and protein levels of ISR-related genes under conditioned culture conditions. Initially, we observed elevated mRNA levels of ATF3 and IDH1, alongside decreased levels of IDH2 and ATF4, after longer conditioned culture periods (Figure 6A). To eliminate the potential antagonistic effects that may occur within cells over time, we decreased the conditioned culture time and again measured the mRNA levels of ISR-related genes in NK cells. After only 1 h of conditioned culture, the mRNA levels of ATF3 and GADD34 were significantly increased, whereas the mRNA levels of ATF4, CHOP, IDH2 and IDH1 were not significantly changed (Figure 6B). We subsequently extracted protein from NK cells in the control group and the conditioned culture group after 2, 4, and 8 hours for Western blot analysis to verify the changes in ISR-related protein levels in NK cells after entry into the OC TME (Figure 6C, Figure S6D). Within 8 hours of conditioned culture, the expression levels of EIF2α, NRF2, and KEAP1 in NK cells did not change significantly. However, the expression of CHOP and ATF4 decreased within 2 hours of conditioned culture, whereas the levels of phosphorylated EIF2α-S51 and ATF3 increased significantly after 4 hours of conditioned culture. This pattern indicates that in NK cells in the TME of OC, the ATF3-mediated ISR is initiated before ferroptosis occurs. To further determine the sequence of ISR initiation in NK cells after entry into the OC TME, we increased and subdivided the conditioned culture time. The Western blot results revealed that the levels of ATF3 and phosphorylated EIF2α-S51 gradually increased, whereas those of EIF2α, CHOP, and ATF4 did not change significantly (Figure 6D), further confirming that an ISR centered on ATF3 rather than ATF4 is initiated in NK cells immediately after entry into the OC TME. In addition, G6PD expression did not change significantly, and IDH2 expression decreased only after 48 hours of conditioned culture (Figure S6E), indicating that G6PD and IDH2 may not be the main factors contributing to the ferroptosis of NK cells.

It has been reported that the ATF4-centered ISR can promote ferroptosis resistance by increasing the transcription of NRF2 through the expression of CHAC1, which is a key activating enzyme of NRF2 31. Moreover, some studies have reported that the activation of ATF3 can inhibit the transcription of NRF2 32-34. On the basis of previous research results and the above data, NK cells are depleted in OC because when they enter the TME, in which the ROS level is high, they induce an ISR centered on ATF3 rather than ATF4, which leads to the inhibition of NRF2 and renders them unable to resist ferroptosis. Given the complex role of the ISR in maintaining cellular viability, we conducted rescue experiments using both the ISR inhibitor ISRIB and the ISR activator CCT020312. Pretreatment with the ISR inhibitor ISRIB reduced the lipid peroxidation level (Figure 6E, Figure S6F) and the proportion of dead cells (Figure 6G, Figure S6H) in NK cells under conditioned culture, whereas pretreatment with the ISR activator CCT020312 had no such effects (Figure 6F, 6H; Figure S6G, S6I), indicating that ISRIB can inhibit the ferroptosis of NK cells in the OC TME.

To verify the ability of ISRIB to increase the tumoricidal effect of NK cells at the animal level, we established abdominal OC tumors in mice and treated the mice with ISRIB and NK cells alone or in combination. The *in vivo* imaging results revealed that the tumor burden in both the ISRIB monotherapy group and the NK cell monotherapy group was lower than that in the untreated control group. Compared with that in the monotherapy group, the tumor burden in the ISRIB-pretreated NK cell-treated group and the ISRIB/NK cell combination therapy group was reduced. Among the groups, the combination therapy group had the lowest tumor burden (Figure 6I), indicating that treatment with ISRIB combined with NK cell therapy had the strongest cytotoxic effect on OC cells. Furthermore, assessment of NK cell infiltration in tumor lesions revealed a substantial increase in the ISRIB-pretreated NK cell therapy group compared with the NK cell monotherapy group (Figure 6J), demonstrating that ISRIB pretreatment significantly enhances NK cell infiltration into the OC tumor core. There was no significant weight loss in the treated group of mice, indicating that the treatment was safe (Figure S6J). Combination treatment with ISRIB and NK cells solves the dilemma of the dysfunctional combination observed for the previously tested ferroptosis inhibitors and NRF2 activators, and the combination of ISRIB and NK cells exerts a triple-synergistic effect to promote OC cell death.

## Discussion

Previous studies have typically attributed NK cell dysfunction to inhibitory cytokines, such as migration inhibitory factors or the dense glycocalyx on the surface of tumor cells; researchers have attempted to modify NK cells to increase their activation and functionality. However, a major obstacle to the efficacy of NK cell therapies in solid tumors is their high propensity to undergo cell death the TME; therefore, our study aimed to investigate the mechanisms of NK cell depletion in the TME and to identify methods that could effectively facilitate NK cell survival in the TME and enhance their antitumor efficacy. We found that NK cell depletion in the TME of OC was due to the occurrence of NRF2 downregulation-mediated ferroptosis. Emerging research increasingly identifies dysregulated lipid metabolism, oxidative stress, and ferroptosis as central regulators of cellular fate within the TME 35. For instance, polyunsaturated fatty acids selectively accumulate in mucosal-associated invariant T cells in metabolic dysfunction-associated steatotic liver disease, driving their ferroptosis 36. In addition, the distinct roles of disulfidptosis and ferroptosis in driving CD8+ T cell exhaustion were revealed 37. It is also reported that iron chelator deferiprone could reprogram OC cells to activate NK cells 38. To address the intertwined role of ferroptosis in antitumor and immunosuppression, a number of novel targeted drugs have been developed, such as ferrous selenide half-shell-covered gold 39, BIN1-targeted therapies 40, homologous magnetic targeted immune vesicles 41 and so on.

The TME has high levels of ROS, which is a common upstream mechanism for both ferroptosis and ISR; however, few studies have investigated the relationship between ferroptosis and ISR, with even fewer studies using immune cells as subjects; thus, we are interested in whether ISR occurs in NK cells in the TME and in its relationship with ferroptosis. We found that NK cells in the TME activate an ISR centered on ATF3 rather than ATF4. On the basis of previous findings that the activation of ATF3 leads to the downregulation of NRF2, we conclude that NK cells in the TME undergo ferroptosis due to the occurrence of an ISR centered on ATF3, which leads to the downregulation of NRF2. Given that ISR occurs in both NK cells and tumor cells in the TME but results in opposite outcomes, we propose a combination therapy using the ISR inhibitor ISRIB with NK cells, which not only synergistically kills OC cells but also enhances the tumor killing effect of NK cells by supporting their survival in the TME, providing a new strategy to improve the efficacy of adoptive cell immunotherapy for solid tumors. The original purpose of ISR activation is to help cells survive, but why ISR centered on ATF3 in NK cells in the TME of OC leads to ferroptosis and whether ISR activation in NK cells is active or passive, as well as the reasons for the selective activation of ATF3 and ATF4, are still unclear. These unresolved questions may help deepen our understanding of the process of tumor immunity. In addition, there is also a paradox between the survival and activation of NK cells in the TME. mTOR activation is thought to be critical for maintaining NK cell proliferation and cytotoxicity 42, whereas another study has shown that the hypoxic environment in cancer leads to sustained activation of the mTOR-Drp1 pathway, which triggers mitochondrial dysfunction in infiltrating NK cells 43. Some researchers have hypothesized that increasing HIF-1α expression in NK cells promotes a metabolic shift from oxidative phosphorylation to glycolysis 44, which may help NK cells overcome hypoxia-mediated apoptosis; however, it is still unknown whether NK cells stably expressing HIF-1α have tumor-killing functions. These seemingly contradictory findings have triggered a more complex discussion about the survival and functional execution of NK cells in the TME; notably, methods for supporting NK cell survival in the TME while providing the correct metabolic mode for their activation are needed.

This study progressively revealed key targets for effectively maintaining NK cell survival in the TME by probing the mechanism of NK cell depletion in a typical immunosuppressive solid tumor, OC, and proposes that combination with the ISR inhibitor ISRIB is the key to improving the efficacy of adoptive NK cell therapy in solid tumors. However, this study has multiple limitations. The combined anticancer therapeutic effects of ISRIB and NK cells need to be validated across a broader spectrum of cancer types. Moreover, the limited number of clinical samples used in this study and the lack of integration with immune checkpoint blockade highlight the need for additional data to support the clinical translation of our findings.

## Supplementary Material

Supplementary figures and tables.
