# Supplementary material for: Overcoming Ferroptosis-Induced Exhaustion of NK Cells through Inhibition of the ATF3-Mediated Integrated Stress Response in Ovarian Cancer
Source: Int J Biol Sci. 2025 Aug 30;21(12):5531–46. doi: 10.7150/ijbs.112615 (PMC12435575; doi:10.7150/ijbs.112615)
Supplement: Supplementary file 1 — Supplementary figures and tables. [file ijbsv21p5531s1.pdf]

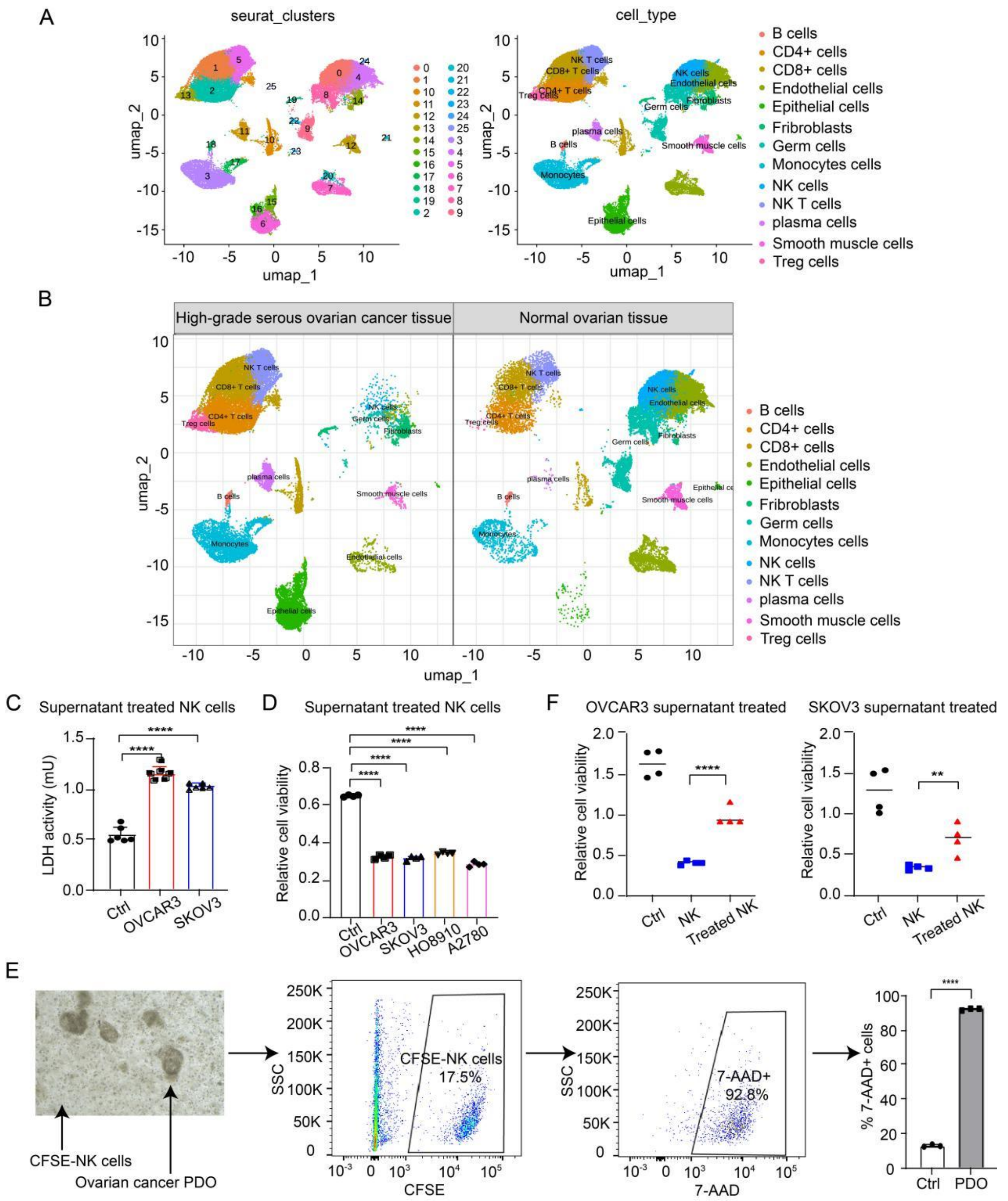

Figure S1, related to Figure 1 Low NK cell infiltration in the TME of OC is due to decreased NK cell viability.

(A) Cell clustering and annotation results of GSE184880 dataset.

(B) Cluster distribution maps of various cells in high-grade serous ovarian cancer tissue group (n = 7) and normal ovarian tissue group (n = 5).

(C) Lactate dehydrogenase activity released by NK cells after 36 hours of conditional culture with the supernatants from OVCAR3 and SKOV3 cell culture media, n = 6.

(D) The activity of NK cells after 36 hours of conditional culture with the supernatants from OVCAR3, SKOV3, HO8910, and A2780 cell culture media, n = 4.

(E) Proportion of 7AAD-positive CFSE-labeled NK cells after directly co-cultured with OC patient-derived organoids for 36 hours, n = 3.

The 24-hour cytotoxic ability of NK cells against the corresponding ovarian cancer cells after 12 hours of conditional culture with the supernatants from OVCAR3 and SKOV3 cell culture media, n = 4. The error bars represent the SEM.

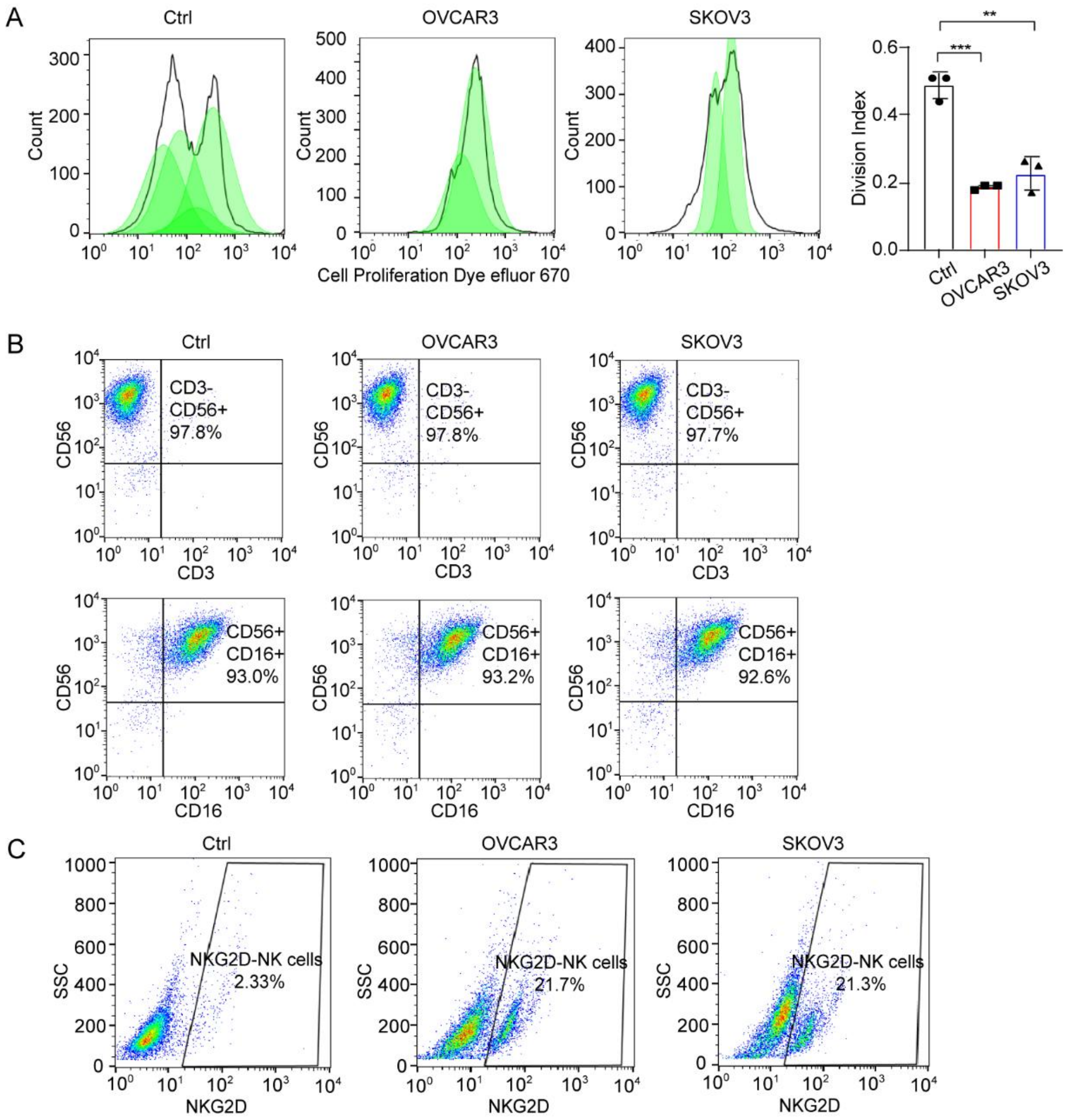

Figure S2, related to Figure 1 Low NK cell infiltration in the TME of OC is due to decreased NK cell viability.

(A) The proliferation of NK cells after 36 hours of conditional culture with the supernatants from OVCAR3 and SKOV3 cell culture media, n = 3.

(B) The grouping situation of NK cells after 36 hours of conditional culture with the supernatants from OVCAR3 and SKOV3cell culture media, n = 1.

(C) The expression of NKG2D on the surface of NK cells after 36 hours of conditional culture with the supernatants from OVCAR3 and SKOV3 cell culture media, n = 1. The error bars represent the SEM.

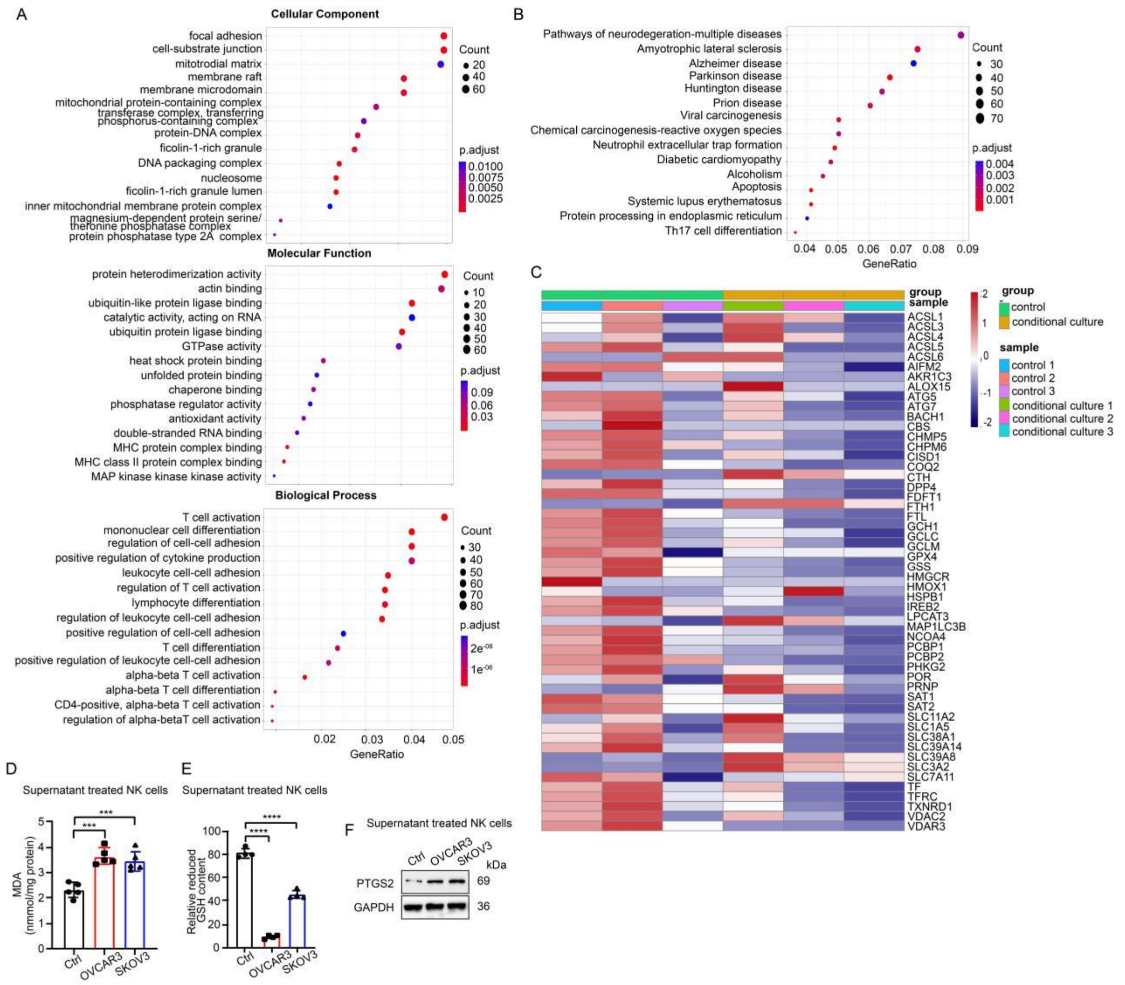

Figure S3, related to Figure 2 NK cells entering the TME of OC undergo ferroptosis.

(A) KEGG functional enrichment of differentially expressed genes.

(B) GO functional enrichment of differentially expressed genes.

(C) Expression heatmaps of ferroptosis pathway related genes in the control and conditional culture groups, n = 3.

(D) The amount of MDA of NK cells after conditional culture with the supernatants from OVCAR3 and SKOV3 cell culture media for 36 hours, n = 3.

(E) The relative reduced GSH content of NK cells after conditional culture with the supernatants from OVCAR3 and SKOV3 cell culture media for 36 hours, n = 3.

(F) The expression of PTGS2 of NK cells after conditional culture with the supernatants from OVCAR3 and SKOV3 cell culture media for 36 hours. The error bars represent the SEM.

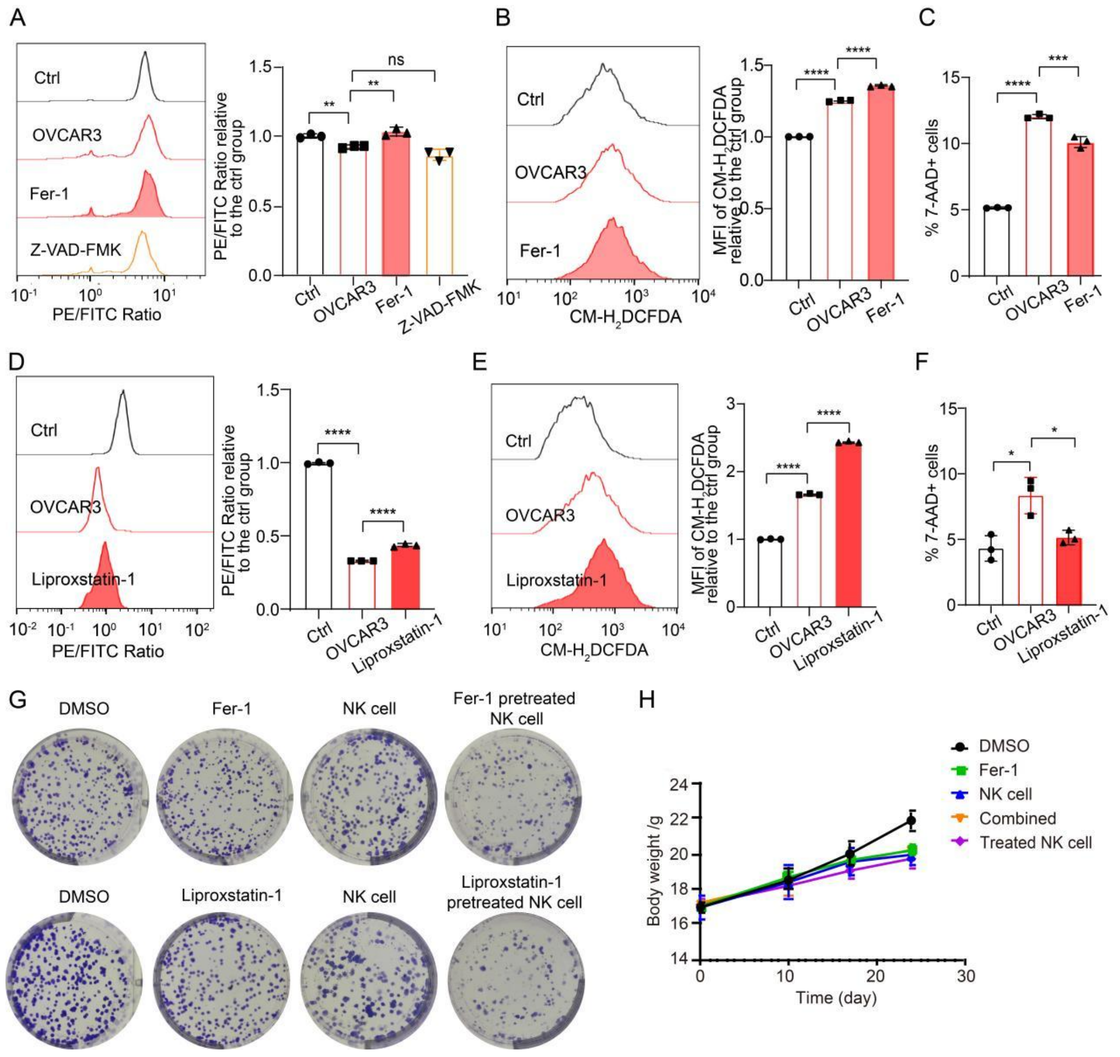

Figure S4, related to Figure 3 Ferroptosis inhibitors inhibit the ferroptosis of NK cells and restore their tumoricidal effect.

(A) Lipid peroxidation level in NK cells after 36 hours of conditional culture with the supernatants from OVCAR3 cell culture media, following pretreatment with Fer-1 (10μM) and Z-VAD-FMK (10μM) for 4 hours, n = 3.

(B) ROS level in NK cells after 36 hours of conditional culture with the supernatants from OVCAR3 cell culture media, following pretreatment with Fer-1 (10μM) for 4 hours, n = 3.

(C) Proportion of 7AAD-positive NK cells after 36 hours of conditional culture with the supernatants from OVCAR3 cell culture media, following pretreatment with Fer-1(10μM) for 4 hours, n = 3.

(D) Lipid peroxidation level in NK cells after 36 hours of conditional culture with the supernatants from OVCAR3 cell culture media, following pretreatment with Liproxstatin-1 (10μM) for 4 hours, n = 3.

(E) ROS level in NK cells after 36 hours of conditional culture with the supernatants from OVCAR3 cell culture media, following pretreatment with Liproxstatin-1 (10μM) for 4 hours, n = 3.

(F) Proportion of 7AAD-positive NK cells after 36 hours of conditional culture with the supernatants from OVCAR3 cell culture media, following pretreatment with Liproxstatin-1 (10μM) for 4 hours, n = 3.
(G) The impact of NK cells, ferroptosis inhibitors and NK cells pre-treated with ferroptosis inhibitors on the colony formation ability of OVCAR3 cells. Fer-1, 10μM; Liproxstatin-1, 10μM.
(H) Body weight of mice on day 0, day 10, day 17, and day 24 since intraperitoneal injection of SKOV3 cells, n = 4. The error bars represent the SEM.

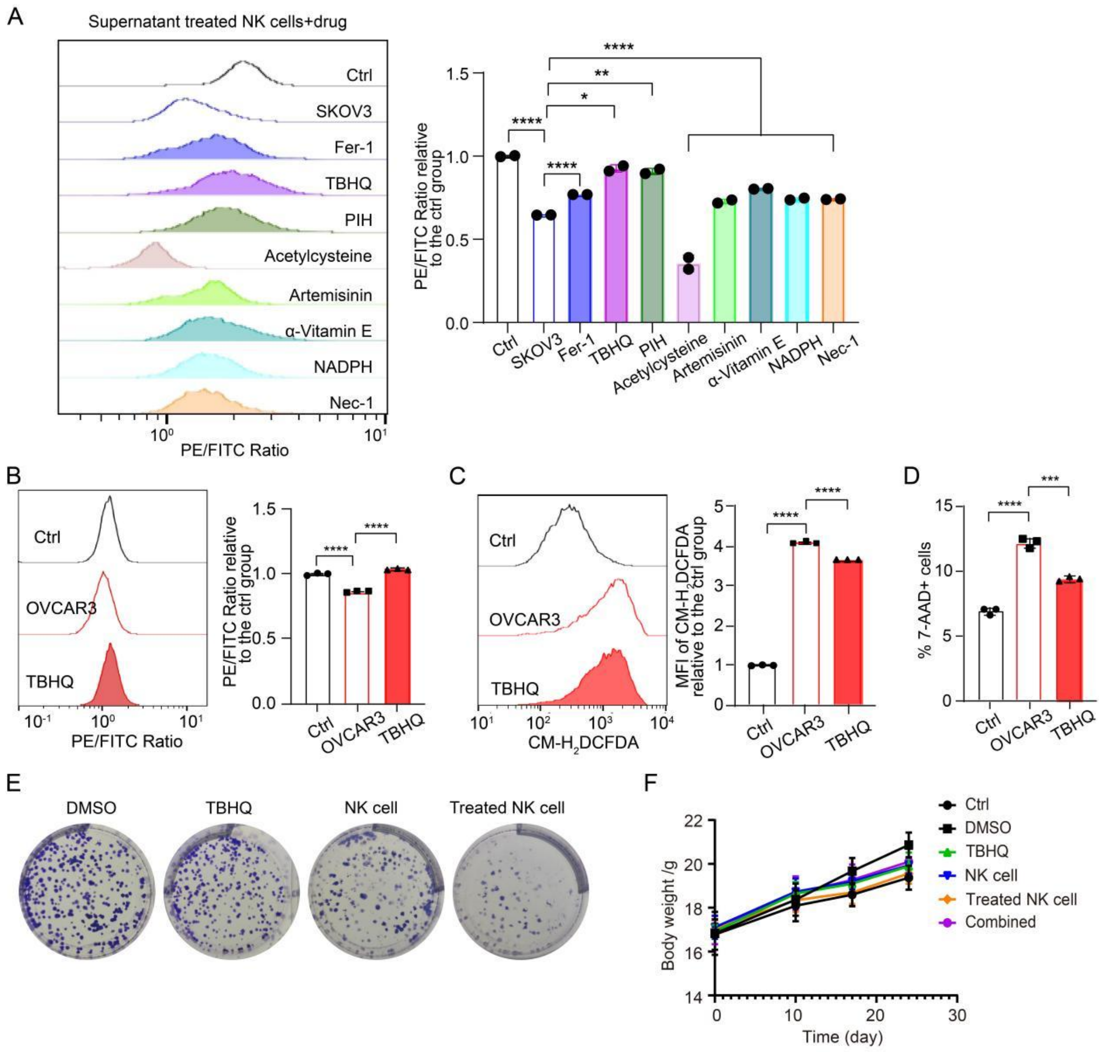

Figure S5, related to Figure 5 TBHQ inhibits the ferroptosis of NK cells in the TME of OC and enhances their cytotoxic effects against OC cells.

(A) Lipid peroxidation level in NK cells after 36 hours of conditional culture with the supernatants from SKOV3 cell culture media, following pretreatment with various drugs for 4 hours, n = 2. Fer-1, 10 μM; TBHQ, 5 μM; PIH, 5 μM; Acetylcysteine, 5 mM; Artemisinin, 10 μM; α-Vitamin E, 50μM; NADPH, 10 μM; Nec-1, 10 μM.

(B) Lipid peroxidation level in NK cells after 36 hours of conditional culture with the supernatants from OVCAR3 cell culture media, following pretreatment with TBHQ (5 μM) for 4 hours, n = 3.

(C) ROS level in NK cells after 36 hours of conditional culture with the supernatants from OVCAR3 cell culture media, following pretreatment with TBHQ (5 μM) for 4 hours, n = 3.

(D) Proportion of 7AAD-positive NK cells after 36 hours of conditional culture with the supernatants from OVCAR3 cell culture media, following pretreatment with TBHQ (5 μM) for 4 hours, n = 3.

(E) The impact of NK cells, TBHQ and NK cells pre-treated with TBHQ on the colony formation ability of OVCAR3 cells.

(F) Body weight of mice on day 0, day 10, day 17, and day 24 since intraperitoneal injection of SKOV3 cells, n = 3. The error bars represent the SEM.

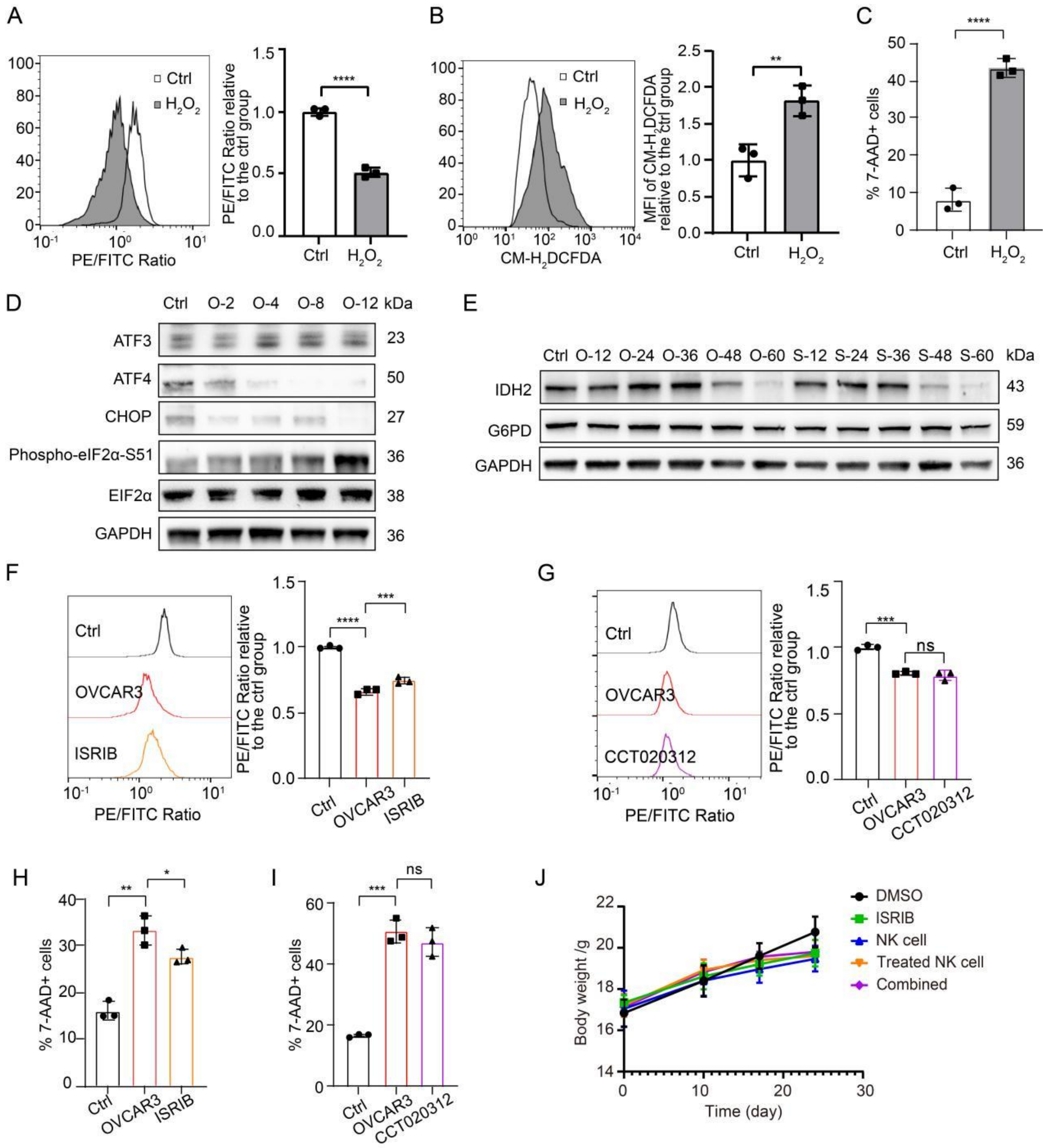

Figure S6. related to Figure 6 High levels of ROS in the TME of OC activate the ATF3-mediated ISR and lead to the ferroptosis of NK cells.

(A) Lipid peroxidation level in NK cells after 36 hours of H_2O_2 (100 μM) treatment, n = 3.
(B) ROS level in NK cells after 36 hours of H_2O_2 (100 μM) treatment, n = 3.
(C) The proportion of 7-AAD positive group of NK cells after 36 hours of H_2O_2 (100 μM) treatment, n = 3.
(D) Differences in protein expression of ISR-related genes in NK cells between the control group and the group conditional cultured with the supernatants from OVCAR3 cell culture media for 2, 4, 8 and 12 hours.
(E) Differences in protein expression of IDH2 and G6PD in NK cells between the control group and the group conditional cultured with the supernatants from OVCAR3 and SKOV3 cell culture media for 12, 24, 36, 48 and 60 hours.

(F) Lipid peroxidation level in NK cells after 36 hours of conditional culture with the supernatants from OVCAR3 cell culture media, following pretreatment with ISRIB (200 nM) for 4 hours, n = 3.

(G) Lipid peroxidation level in NK cells after 36 hours of conditional culture with the supernatants from OVCAR3 cell culture media, following pretreatment with CCT020312 (10 μM) for 4 hours, n = 3.

(H) Proportion of 7AAD-positive NK cells after 36 hours of conditional culture with the supernatants from OVCAR3 cell culture media, following pretreatment with ISRIB (200 nM) for 4 hours, n = 3.

(I) Proportion of 7AAD-positive NK cells after 36 hours of conditional culture with the supernatants from OVCAR3 cell culture media, following pretreatment with CCT020312 (10 μM) for 4 hours, n = 3.

(J) Body weight of mice on day 0, day 10, day 17, and day 24 since intraperitoneal injection of SKOV3 cells, n = 3. The error bars represent the SEM.

Table S1: Primary antibodies

Target protein	Clonality	Species	Company	Product #	Dilution	Use
CD3	monoclonal	mouse	Proteintech	60181-1-Ig	1:200	IF
CD68	monoclonal	mouse	Proteintech	66231-2-Ig	1:3000	IF
CD56	monoclonal	mouse	Proteintech	60238-1-Ig	1:200	IF
CD16	monoclonal	mouse	Proteintech	66779-1-Ig	1:200	IF
eFluor™ 506 Anti-Human CD3	monoclonal	mouse	eBioscience	69-0038-41	5μL/test	FCM
647 Anti-Human CD56	monoclonal	mouse	Proteintech	CL647-65264	5μL/test	FCM
FITC Anti-Human CD16	monoclonal	mouse	eBioscience	11-0168-41	5μL/test	FCM
eBioscience™ CFSE	/	/	eBioscience	65-0850-84	5μL/test	FCM
7-AAD Viability Staining Solution	/	/	eBioscience	00-6993-50	5μL/test	FCM
eFluor™ 670 Cell Proliferation Dye	/	/	eBioscience	65-0840-90	5μM	FCM
PE anti-human NKG2D	monoclonal	mouse	Biolegend	320805	5μL/test	FCM
FITC anti-human Granzyme B	monoclonal	mouse	Biolegend	515403	5μL/test	FCM
PE anti-human CD107a	monoclonal	mouse	eBioscience	12-1079-42	5μL/test	FCM
eFluor™ 450 IFNγ	monoclonal	mouse	eBioscience	48-7319-41	5μL/test	FCM
PE anti-human CXCR3	monoclonal	mouse	eBioscience	12-1839-42	5μL/test	FCM
PE anti-human CXCR1	monoclonal	mouse	eBioscience	12-1819-41	5μL/test	FCM
GAPDH	monoclonal	mouse	Origene	TA802519	1:2000	WB
PTGS2	monoclonal	rabbit	ABclonal	A3560	1:1000	WB
GPX4	monoclonal	rabbit	ABclonal	A11243	1:1000	WB
SLC7A11	monoclonal	rabbit	ABclonal	A2413	1:500	WB
NRF2	monoclonal	rabbit	ABclonal	A21176	1:500/1:200	WB/IF
KEAP1	monoclonal	rabbit	ABclonal	A25297	1:1000	WB
ACSL3	monoclonal	rabbit	ABclonal	A22085	1:1000	WB
LPCAT3	polyclonal	rabbit	ABclonal	A17604	1:500	WB
ACSL4	monoclonal	rabbit	ABclonal	A20414	1:10000	WB
DMT1	monoclonal	rabbit	ABclonal	A23379	1:500	WB
NCOA4	polyclonal	rabbit	ABclonal	A25307	1:500	WB
FTH1	monoclonal	rabbit	ABclonal	A19544	1:1000	WB
Bax	monoclonal	rabbit	Abcam	ab32503	1:2000	WB
TFRC	monoclonal	rabbit	ABclonal	A22161	1:10000	WB
Lamin B1	monoclonal	rabbit	Proteintech	66095-1-Ig	1:20000	WB
Alpha Tubulin	monoclonal	rabbit	Proteintech	66031-1-Ig	1:20000	WB
EIF2α	monoclonal	rabbit	ABclonal	A21221	1:2000	WB
p-EIF2α	monoclonal	rabbit	ABclonal	AP0692	1:1000	WB
CHOP	monoclonal	rabbit	ABclonal	A20987	1:500	WB
ATF4	polyclonal	rabbit	ABclonal	A0201	1:500	WB
ATF3	polyclonal	rabbit	ABclonal	A13469	1:500	WB
IDH2	monoclonal	rabbit	ABclonal	A11270	1:500	WB
G6PD	monoclonal	rabbit	ABclonal	A11234	1:1000	WB

Table S2: sequences

Primers	Species	sequences
GAPDH	Human	Forward: 5’-GGAGCGAGATCCCTCCAAAAT-3’<br>Reverse: 5’-GGCTGTTGTCATACTTCTCATGG-3’
SLC7A11	Human	Forward: 5’-TCTCCAAAGGAGGTTACCTGC-3’<br>Reverse: 5’-AGACTCCCCTCAGTAAAGTGAC-3’
NRF2	Human	Forward: 5’-TTCCCGGTCACATCGAGAG-3’<br>Reverse: 5’-TTCCCGGTCACATCGAGAG-3’
CHAC1	Human	Forward: 5’-GAACCCTGGTTACCTGGGC-3’<br>Reverse: 5’-CGCAGCAAGTATTCAAGGTTGT-3’
ACSL3	Human	Forward: 5’-CGCAGCAAGTATTCAAGGTTGT-3’<br>Reverse: 5’-CTATGAGGTTGGTTTTCCATGCT-3’
LPCAT3	Human	Forward: 5’-GGCTGGATACTATTACACTGCC-3’<br>Reverse: 5’-GATCTTTCCCTCCGTCAAAGTAG-3’
ACSL4	Human	Forward: 5’-CATCCCTGGAGCAGATACTCT-3’<br>Reverse: 5’-TCACTTAGGATTTCCCTGGTCC-3’
FAR1	Human	Forward: 5’-CACGCCAAAATTGATAGGAGACA-3<br>Reverse: 5’-CCTGCCGCAATAAAGAGACC-3’
DMT1	Human	Forward: 5’-ATCGGCTCAGACATGCAAGAA-3’<br>Reverse: 5’-TTCCGCAAGCCATATTTGTCC-3’
NCOA4	Human	Forward: 5’-ACAGTTGCATAAGCCGTCACC-3’<br>Reverse: 5’-TGAGCCTGCTGTTGAAGTGTC-3’
ACO1	Human	Forward: 5’-AACCCATTCGCACACCTTG-3’<br>Reverse: 5’-ATGGTAAGCGCCCATATCTTG-3’
ATG7	Human	Forward: 5’-ATGATCCCTGTAACTTAGCCCA-3’<br>Reverse: 5’-CACGGAAGCAAACAACTTCAAC-3’
ATF3	Human	Forward: 5’-CCTCTGCGCTGGAATCAGTC-3’<br>Reverse: 5’-TTCTTTCTCGTCGCCTCTTTTT-3’
ATF4	Human	Forward: 5’-CTCCGGGACAGATTGGATGTT-3’<br>Reverse: 5’-GGCTGCTTATTAGTCTCCTGGAC-3’
IDH1	Human	Forward: 5’-AGAAGCATAATGTTGGCGTCA-3’<br>Reverse: 5’-CGTATGGTGCCATTTGGTGATT-3’
IDH2	Human	Forward: 5’-CCCGTATTATCTGGCAGTTCATC-3’<br>Reverse: 5’-ATCAGTCTGGTCACGGTTTGG-3’
CHOP	Human	Forward: 5’-GGAAACAGAGTGGTCATTCCC-3’<br>Reverse: 5’-CTGCTTGAGCCGTTCATTCTC-3’
GADD34	Human	Forward: 5’-ATGATGGCATGTATGGTGAGC-3’<br>Reverse: 5’-AACCTTGCAGTGTCCTTATCAG-3’
